# Extremely Scalable Spiking Neuronal Network Simulation Code: From Laptops to Exascale Computers

**DOI:** 10.3389/fninf.2018.00002

**Published:** 2018-02-16

**Authors:** Jakob Jordan, Tammo Ippen, Moritz Helias, Itaru Kitayama, Mitsuhisa Sato, Jun Igarashi, Markus Diesmann, Susanne Kunkel

**Affiliations:** ^1^Institute of Neuroscience and Medicine (INM-6) and Institute for Advanced Simulation (IAS-6) and JARA Institute Brain Structure-Function Relationships (INM-10), Jülich Research Centre, Jülich, Germany; ^2^Faculty of Science and Technology, Norwegian University of Life Sciences, Ås, Norway; ^3^Department of Physics, Faculty 1, RWTH Aachen University, Aachen, Germany; ^4^Advanced Institute for Computational Science, RIKEN, Kobe, Japan; ^5^Computational Engineering Applications Unit, RIKEN, Wako, Japan; ^6^Department of Psychiatry, Psychotherapy and Psychosomatics, Medical Faculty, RWTH Aachen University, Aachen, Germany; ^7^Department of Computational Science and Technology, School of Computer Science and Communication, KTH Royal Institute of Technology, Stockholm, Sweden; ^8^Simulation Laboratory Neuroscience – Bernstein Facility for Simulation and Database Technology, Jülich Research Centre, Jülich, Germany

**Keywords:** supercomputer, large-scale simulation, parallel computing, spiking neuronal network, exascale computing, computational neuroscience

## Abstract

State-of-the-art software tools for neuronal network simulations scale to the largest computing systems available today and enable investigations of large-scale networks of up to 10 % of the human cortex at a resolution of individual neurons and synapses. Due to an upper limit on the number of incoming connections of a single neuron, network connectivity becomes extremely sparse at this scale. To manage computational costs, simulation software ultimately targeting the brain scale needs to fully exploit this sparsity. Here we present a two-tier connection infrastructure and a framework for directed communication among compute nodes accounting for the sparsity of brain-scale networks. We demonstrate the feasibility of this approach by implementing the technology in the NEST simulation code and we investigate its performance in different scaling scenarios of typical network simulations. Our results show that the new data structures and communication scheme prepare the simulation kernel for post-petascale high-performance computing facilities without sacrificing performance in smaller systems.

## 1. Introduction

Modern neuroscience has established numerical simulation as a third pillar supporting the investigation of the dynamics and function of neuronal networks, next to experimental and theoretical approaches. Simulation software reflects the diversity of modern neuroscientific research with tools ranging from the molecular scale to investigate processes at individual synapses (Wils and De Schutter, 2009) to whole-brain simulations at the population level that can be directly related to clinical measures (Sanz Leon et al., 2013). Most neuronal network simulation software, however, is based on the hypothesis that the main processes of brain function can be captured at the level of individual nerve cells and their interactions through electrical pulses. Since these pulses show little variation in shape, it is generally believed that they convey information only through their timing or rate of occurrence. Simulators in this area that follow a general-purpose approach employ simplified models of neurons and synapses with individually configurable parameters and connectivity (Carnevale and Hines, 2006; Bower and Beeman, 2007; Gewaltig and Diesmann, 2007; Bekolay et al., 2013; Goodman and Brette, 2013). In such simplified models, individual neuron and synapse dynamics are typically described by a small number of coupled differential equations. Besides, in order to address models of learning in neuronal networks most simulators support a variety of plasticity mechanisms such as short- and long-term plasticity (Morrison et al., 2008), neuromodulated plasticity (Potjans et al., 2010) and structural plasticity (Diaz-Pier et al., 2016).

It is widely believed that high-level brain function is not solely the product of complex dynamics of isolated brain areas, but involves coordinated interaction between multiple cortical and subcortical areas (Kandel et al., 1991; Bressler and Menon, 2010). To gain insights into cortical information processing, large-scale network models aim to account for several areas and their interactions involving millions of neurons and billions of synapses. A systematic reduction in neuron and synapse density compared to biological tissue is severely limited as soon as researchers strive to faithfully represent even just pairwise coordinated neuronal activity (van Albada et al., 2015), making the need for full-scale models apparent. While workstations allow simulations of up to 10^5^ neurons, corresponding to the number of neurons under approximately 1 mm^2^ of cortical surface, larger networks require distributed simulations (Senk et al., 2015; Schmidt et al., 2016). State-of-the-art simulation software allows researchers to simulate about 10 % of the human cortex at a resolution of individual neurons and synapses on contemporary supercomputers (Kunkel et al., 2014). For these large-scale simulations one of the main computational challenges is the high connectivity of neuronal networks. The human cortex consists of about 10^10^ cells, each receiving about 10^4^ connections (Abeles, 1991; Stepanyants et al., 2009), which leads to an estimated 10^14^ synapses. Representing each of the connections by two double precision numbers requires about 1.6 PB of main memory. To complicate things further, neurons receive only about 50 % of their connections from other nerve cells in their vicinity, while the remainder are long-range connections from various remote areas (Abeles, 1991; Braitenberg and Schüz, 1991). This feature distinguishes neuronal simulations from simulations of classical physical systems, for example by finite-element methods exploiting the locality of physical interactions (see, e.g., Johnson, 1987), and poses severe difficulties for dynamic load balancing: One cannot easily move computational units from one process to the next without major changes to the fundamental data structures.

Over a decade ago simulation codes started to store the data that represent synapses exclusively on the compute node where the target neuron resides (Morrison et al., 2005), in the following referred to as the postsynaptic side. For typical neuronal network models this approach enables parallel network construction with none or little communication between the compute nodes. Furthermore, in this scheme only the occurrence of a spike in a particular source neuron needs to be transmitted to other compute nodes, often in source-based address-event-representation (AER; Boahen, 2000; Lansner and Diesmann, 2012). Typically all spikes generated in the network are gathered on each compute node (Hines et al., 2011). Postsynaptic data structures are then responsible for obtaining the relevant synapse parameters from local memory and routing the spike to the correct target. To distribute workload evenly across compute nodes also for highly structured networks with heterogeneous population properties, neurons are distributed across compute nodes in a round-robin fashion. On small machines the number of compute nodes participating in a simulation is smaller than the number of synapses per neuron such that each neuron typically has many targets on every compute node. In this setting it is efficient to maintain on each compute node a resizable array containing references to local target lists with one element for each neuron in the network that could be indexed by the source neuron's identifier (Morrison et al., 2005). With growing network size and the availability of a new generation of supercomputers, the ratio between the number of compute nodes and the number of synapses per neuron reversed. This led to a replacement of the resizable array by a sparse table with subsequent target lists implemented as dynamic container types that have little overhead for a small number of local targets. The sparse table accounts for the sparsity of large-scale networks by consuming only few bits of memory for each empty entry (Kunkel et al., 2014). These data structures enabled simulation codes that perform equally well on small- to large-scale simulations, and scale well to the largest supercomputers available today (“petascale” regime, 10^15^ FLOPS) (Kunkel et al., 2014). Over the next decade, exascale machines will be developed that will most likely show only a moderate increase in the number of compute nodes but a significant increase in the number of threads and amount of memory per compute node (see, e.g., Dongarra et al., 2011). If neural simulators are able to fully exploit the computational power of these new machines, researchers, for the first time, will be able to simulate the full human cortex at cellular resolution.

**Figure 1:**
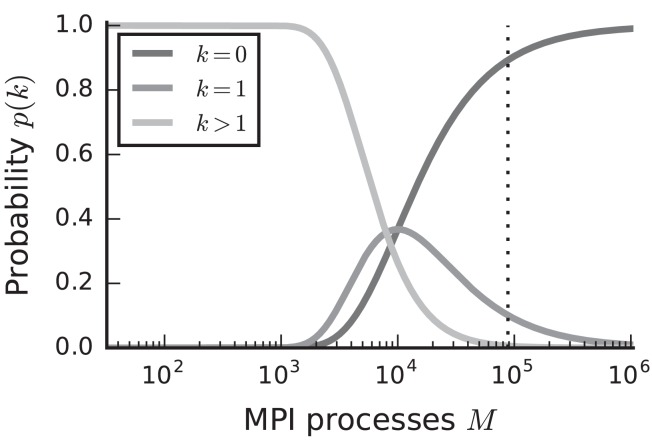
Probability of a model neuron to have a certain number of targets on a randomly selected process in a weak scaling scenario. Probability of a neuron to have zero (dark gray), one (medium gray), or more (light gray) targets. Vertical dashed line marks maximal number of MPI processes, also called ranks, on the K computer. Targets per neuron *K* = 10^4^, number of MPI processes *M* ∈ {32; …; 1, 048, 575}. This number of connections is typical for a pyramidal cell in cortex (Abeles, 1991).

In the case of purely postsynaptic storage of connection information, all spikes from the network are collected on all compute nodes since only postsynaptic data structures are responsible for routing the spikes to the correct targets. To support efficient delivery of spikes, this requires the postsynaptic data structures to store at least one bit per neuron in the network signaling the presence or absence of local targets (Kunkel et al., 2014). In this case memory usage per compute node scales with the total number of neurons in the network, consuming a major part of the available local memory from about 10^9^–10^10^ neurons on. However, networks in which each neuron receives a fixed number of connections become extremely sparse at this scale. In the following we assume a distributed setting in which each compute node is running a single instance of the simulator and that these instances communicate via the Message Passing Interface (MPI). As customary, we refer to a single such instance also as an “MPI process” or “rank” (Message Passing Interface Forum, 2009). Assuming a fixed number of neurons per process and 10^4^ targets per neuron, most neurons have multiple targets on every process for less than 10^3^ MPI processes (Figure 1, light gray). This probability quickly decays to zero between 10^3^ and 10^4^ processes as it becomes more likely to find exactly one target per process (Figure 1, medium gray). Beyond 10^4^ MPI processes it is overwhelmingly likely that a specific neuron has no target on a randomly selected process (Figure 1, dark gray), implying that most spikes collected on a compute node do not have any local targets (see also Hines et al., 2011). This estimate demonstrates that a communication scheme that relies purely on postsynaptic routing of spikes cannot be efficient in the regime of tens of thousands of MPI processes. Since each process needs to check the existence of local targets for each spike, the total runtime increases proportionally to the total number of spikes generated in the network and hence with the total network size, assuming constant firing rates per neuron. Hence, for large networks this is a large contribution to the total runtime of the simulation (cf. Schenck et al., 2014). Nevertheless, the probability that at least one neuron on a randomly selected MPI process has local targets on another randomly selected process, is not small. Consequently all processes potentially need to communicate spikes to each other at some point during a simulation, ruling out communication schemes that only include subsets of nodes.

In this manuscript we describe a two-tier connection infrastructure with a pre- and postsynaptic part that replaces the above mentioned purely postsynaptic storage of connection information and enables directed communication among compute nodes. In particular we replace all data structures that scale proportionally to the total number of neurons in the network or the total number of MPI processes, because they consume significant amounts of memory for simulations of networks of 10^9^ neurons or more. In addition to these improvements, the directed communication of spikes makes sure each MPI process only needs to process the spikes which are required locally. While there is need for large-scale simulations, many researchers investigate small to medium size networks. Therefore, the new data structures and communication framework should not lead to a penalty for simulations run on laptops, workstations, and moderately sized clusters. We consequently introduce additional optimizations to maintain high performance in small- to medium-scale simulations. This approach preserves a common codebase for laptops and HPC systems, reducing maintenance costs and supporting quick adoption of features designed for either use case.

The remainder of this work is organized as follows: Section 2.1 describes an archetypal network model as the main use case and section 2.2 introduces three scaling scenarios. Section 2.3 continues by describing the three supercomputing platforms employed in this study. Section 2.4 gives a short overview of the NEST simulator for which we provide an implementation of the new connection infrastructure and communication scheme. Subsequently section 2.5 adapts a model of memory consumption per compute node to predict the influence of the new data structures. Section 3 first summarizes the limitations of the connection infrastructure implemented in the previous kernel, henceforth referred to as “4g”, and afterwards describes the new two-tier infrastructure and the corresponding network construction procedure of the new simulation kernel (“5g”) in section 3.1. Section 3.2 introduces the new spike exchange method and section 3.3 considers small-scale simulations and corresponding optimizations. Finally section 3.4 and 3.5 discuss the results of the model of memory usage and performance measurements in the scaling scenarios. The study concludes by discussing limitations of the new technology and future extensions (section 4).

The technology described in the present article will be made available to the community with one of the next releases of the open-source simulation software NEST. The conceptual and algorithmic work described here is a module in our long-term collaborative project to provide the technology for neural systems simulations (Gewaltig and Diesmann, 2007).

## 2. Materials and methods

### 2.1. Benchmark network model

To analyze the memory usage of the new data structures (see section 2.5) and to measure the actual memory usage and run time of the implementation (see section 2.2) we employ a balanced random network model with plastic connections, also used in previous publications on neuronal network simulation technology (Helias et al., 2012; Kunkel et al., 2012, 2014; Ippen et al., 2017; Kunkel and Schenck, 2017). The network consists of two recurrently connected populations: one excitatory and one inhibitory, where excitatory neurons outnumber inhibitory neurons by a factor of four. To ensure stability of the network the inhibitory connections are much stronger than the excitatory connections. Neurons are modeled by single-compartment leaky-integrate-and-fire neurons with alpha-shaped postsynaptic currents and have homogeneous parameters within and across the two populations. Connections are drawn randomly for each neuron with a fixed number of incoming connections per neuron independent of the network size. The excitatory-excitatory connections exhibit spike-timing dependent plasticity (STDP, see, e.g., Morrison et al., 2007), while all other connections are static. This model serves as a scalable version of a typical neuronal network simulation. The results obtained with this particular model generalize, as long as the total memory usage of synapses is significantly larger than that of neurons, and interactions are mainly mediated via chemical synapses. Due to its random connectivity the network model represents a worst-case scenario in terms of network structure: A single neuron projects with equal probability to any other neuron in the network such that local communication patterns cannot be exploited by representing strongly connected subnetworks on a subset of the available compute nodes. A detailed network description and parameter values can be found in section A in the Appendix and a variant of the simulation script is available from the most recent NEST release as hpc_benchmark.sli (Kunkel et al., 2017).

### 2.2. Measuring scalability

To assess the scalability of the simulation code across MPI processes and threads, we investigate three scaling scenarios. For an in-depth discussion of the relevant scaling scenarios and possible pitfalls, see van Albada et al. (2014). A single compute node in a modern HPC system contains tens of cores. In order to make optimal use of the available compute power and limited amount of memory, a lightweight parallelization scheme, like OpenMP (OpenMP Architecture Review Board, 2008), should be used within a single compute node (Ippen et al., 2017). We hence run a single MPI process with multiple OpenMP threads per compute node in all simulations of this study. Accordingly we use “MPI processes” and “compute nodes” interchangeably in this manuscript.

In weak-scaling benchmarks, the problem size per compute node is fixed while the number of compute nodes and thus the total problem size is varied. In our case, we simulate a constant number of neurons per compute node where all neurons have a fixed in-degree, which leads to an increase in network size with sparser connectivity as the number of compute nodes grows. A weak-scaling experiment uncovers limiting factors for scalability in terms of memory usage and runtime that increase proportionally with the total network size (~N) or the number of MPI processes (~M).

In strong-scaling measurements, the total problem size is fixed while the number of MPI processes or threads per process is varied. Here, we fix the total network size including the number of connections. With an increasing number of MPI processes or threads per process, this reduces the load per process or thread, addressing the question of how fast a network of a particular size can be simulated. While in our application a strong-scaling test over MPI processes uncovers communication bottlenecks, a strong-scaling test over threads mainly exposes serial parts of the code.

In addition to weak-scaling and strong-scaling experiments, we perform a maximum-filling scaling. For a given amount of computational resources, in terms of available memory per compute node, we determine the maximal problem size that can be simulated. This is not necessarily identical to a weak scaling if, for example, the memory usage of the application changes with the number of MPI processes. In our case, we determine the maximal number of neurons that just fits into the available memory for a given number of compute nodes where all neurons have a fixed in-degree. Since the maximum network size is difficult to determine, we obtain a prediction from the memory-usage model (see section 2.5) before performing a full-scale run. The maximum-filling scaling scenario tests the limits of the software in terms of memory usage and addresses the issue of efficient use of available computational resources: How many compute nodes does a specific network simulation require at least?

### 2.3. Supercomputers

We run benchmarks on three HPC systems that are commonly employed for (neuro)scientific research: the JUQUEEN BlueGene/Q and JURECA systems at the Jülich Research Centre, Germany, and the K computer at the Advanced Institute for Computational Science in Kobe, Japan.

The JUQUEEN supercomputer (Jülich Supercomputing Centre, 2015) consists of 28,672 compute nodes, each equipped with a 16-core IBM PowerPC A2 processor running at 1.6 GHz and 16 GB RAM, leading to a peak performance of about 5.9 PFLOPS and 448 TB of main memory in total. The communication network is implemented as a 5D torus with a bandwidth of 40 GBps. Applications are compiled using the IBM XL compiler suite. JUQUEEN supports hybrid parallelism, with multithreading via OpenMP within a single compute node and MPI for distributed-memory computing. The GNU Scientific library^1^ is available in version 2.1.

JURECA consists of 1872 compute nodes, each housing two Intel Xeon E5-2680 v3 Haswell CPUs at 2.5 GHz for a total of 1.8 PFLOPS. Most of the compute nodes have 128 GiB of memory available. The system provides 75 compute nodes equipped with two NVIDIA K80 GPUs, which, however, were not used in this study. Nodes are connected via Mellanox EDR InfiniBand. To compile applications, we rely on the GNU Compiler Collection (GCC) and link against the ParaStationMPI library for MPI support.

The K computer (Miyazaki et al., 2012) features 82,944 compute nodes, each with an 8-core SPARC64 VIIIfx processor operating at 2 GHz, with 16 GB RAM/node, leading to a peak performance of about 11.3 PFLOPS and a total of *1377* TB of main memory. The compute nodes are interconnected via the “Tofu” (“torus connected full connection”) network with 5 GBps per link. The K computer supports hybrid parallelism with OpenMP (v3.0) at the single node level and MPI (v2.1) for inter-node communication. Applications are compiled with the Fujitsu C/C++ Compiler.

### 2.4. NEST simulator

NEST is an open-source software tool that is designed for the simulation of large-scale networks of single-compartment spiking neuron models (Gewaltig and Diesmann, 2007). It is developed and maintained by the NEST initiative^2^ under the GNU General Public License, version 2^3^ and can be freely downloaded from the website of the NEST simulator^4^. The collaborative development of NEST follows an iterative, incremental strategy derived from the requirements and constraints given by the community (Diesmann and Gewaltig, 2002). Users can control simulations either via a built-in scripting language (SLI) or a Python module (PyNEST; Eppler et al., 2009; Zaytsev and Morrison, 2014). While the definition of the network, in terms of the specification of neuronal populations and connections, can be conveniently performed in procedural form in an interpreted language, all compute-intensive tasks such as the actual generation of connectivity or the propagation of neuron dynamics are executed by the simulation kernel implemented in C++. NEST supports a wide variety of computing platforms, from laptops to moderately-sized clusters and supercomputers using a common codebase. To optimally use the available compute resources, NEST supports hybrid parallelization employing MPI for inter-node communication and multi-threading via OpenMP within each MPI process. Running multiple threads instead of multiple MPI processes per compute node makes better use of the available memory (Ippen et al., 2017).

Neurons are distributed in a round-robin fashion across all available threads according to their global id (GID), which labels all neurons and devices in the network uniquely by order of creation. The round-robin distribution of neurons implements a simple form of static load balancing as it ensures that neurons which belong to the same population and are hence expected to exhibit similar activity patterns, are evenly distributed across cores. Devices for stimulation and recording, are duplicated on each thread and only send to or record from thread-local neurons to avoid expensive communication of status variables. Events between neurons are communicated between processes by collective MPI functions (see section 3.2). Most data structures are private to each thread within a compute node. This separation is however relaxed during writing of events to MPI buffers and reading of events from the buffers to improve efficiency and reduce serial overhead (see sections 3.1.3 and 3.2). NEST offers a range of neuron and synapse models from low to high complexity. Users can extend the range of available models by employing a domain-specific model description language (Plotnikov et al., 2016) or by providing an appropriate implementation in C++. The simulation kernel supports further biophysical mechanisms, for example neuromodulated plasticity (Potjans et al., 2010), structural plasticity (Diaz-Pier et al., 2016), coupling between neurons via gap junctions (Hahne et al., 2015), and non-spiking neurons with continuous interactions, such as rate-based models (Hahne et al., 2017).

### 2.5. Adaptation of memory-usage model

Improving algorithms and data structures within an existing software project requires first of all identification of the main bottlenecks. This involves measurements of runtime and memory usage (see, e.g., Hager and Wellein, 2011), since any intuitions about possible bottlenecks can be severely misleading (see, e.g., Bentley, 1982). This is ever more the case when redesigning algorithms and data structures that need to scale to tens of thousands of processes. Measurements for large-scale applications, however, consume time and resources. Kunkel et al. (2012) therefore introduce a model that allows the prediction of the memory usage of a neural simulator accounting for contributions from different objects, such as neurons, synapses, and the corresponding infrastructure. The model considers only the leading order contributions to the overall memory consumption and needs to be checked against actual measurements to prove its sufficiency.

The model describes the memory consumption per MPI process as a function of compute environment and network parameters such as the number of MPI processes, the number of threads per process, and the total number of neurons in the network. Applying this model to NEST as a concrete use case allows us to predict the effect of potential changes to code quickly, without running simulations, and hence to target our efforts on critical parts of the codebase. In addition the model enables us to determine the limits, in our case in terms of network size, for a particular amount of compute resources (see section 2.2). In the following we only mention changes to the previous formulation of the memory-usage model arising due to the introduction of a two-tier connection infrastructure. For extensive discussions of the memory-usage model, please refer to Helias et al. (2012); Kunkel et al. (2012). See Kunkel et al. (2014) for the memory-usage model describing the previous simulation kernel.

In the previous simulation kernel, all information about connections is exclusively stored on the postsynaptic side, which is the compute node on which the target neuron resides. The main differences in the memory-usage model for the new kernel are additional terms that describe the memory used for constructing and storing the presynaptic part of the connection infrastructure. Originally, the model was defined as a function of the number of MPI processes *M*, the number of threads per MPI process *T*, the total number of neurons *N* and the average number of connections per neuron *K*. Since we keep *K* fixed throughout this study, we will describe the memory usage as a function of only three variables: M (*M, T,N*). Please refer to section A in the Appendix for the numerical values of all other parameters that appear in the equations below. The total memory usage can be divided into three components: base memory usage and MPI buffers M_0_ (*M, T,N*), memory usage of nodes M_n_ (*M,N*), and memory usage of connections M_c_ (*M, T,N*). The latter two components do not just contain the memory usage of individual neuron and synapse objects, but also contributions from infrastructure needed for efficient access to the individual objects during simulation. This leads to the following definition of the full model (cf. Kunkel et al., 2014):

$$M(M,\;T,N)\;=M_{0}\text{ (}M,\;T,N\text{) }+M_{\text{n}}\text{ (}M,N)\;+M_{\text{c}}\text{ (}M,\;T,N).$$

The first term contains the empirically measured base memory usage M_b_, including the memory required by the simulation kernel just after startup as well as MPI and OpenMP overhead. Furthermore this term additionally captures the memory usage of MPI buffers for the communication data required for constructing the presynaptic part of the two-tier connection infrastructure (see section 3.1.3) and for the spike events during simulation. This leads to the following definition of M_0_:

$$\begin{array}{l} M_{0}\;(M,\;T,N)\;=\;M_{\text{b}}\;+\text{ min}(B_{\text{c}},N_{M}\text{ min}(K,MT))m_{\text{td}}\\ \;+\text{ min}(B_{\text{s}},N_{M}v_{\text{max}}\text{ min}(K,MT))m_{\text{sd}}\;, \end{array}$$

where we introduce the shorthand $N_{M}:=\frac{N}{M}.$ Here *m*_td_ denotes the memory consumption for a single entry in the MPI buffer used for communication of connectivity data (section 3.1.2) and *m*_sd_ denotes the memory consumption for a single entry in the MPI buffer used for communication of spikes (section 3.2). The particular forms of the latter two terms result from the following considerations: Since we employ MPI_Alltoall (explained in section 3), the buffer size for a single communication round must be the same across all MPI processes. If more data need to be communicated than a single communication round can handle, we initiate another round of collective communication and double the size of the respective buffers, up to a user-defined maximal size, denoted by *B*_c_ and *B*_s_, respectively (cf. sections 3.1.3 and 3.2). The (average) number of connections and spikes can be estimated as *N_M_* min(*K, MT*) and *N_M_ν*_max_ min(*K, MT*), respectively, where ν_max_ denotes the maximal firing rate in the network. We assume that a single neuron has an average out-degree of *K*, independent of the size of the network as described in section 2.1 and consistent with biological data (Abeles, 1991). The occurrence of the total number of threads (*MT*) in min(*K, MT*) is due to adaptations for pre-petascale machines as described in section 3.3.

As the redesign affects only the connection infrastructure and communication framework, the contributions of neurons and neuronal infrastructure are the same as in the memory-usage model for the previous kernel. For a definition and discussion of this second contribution M_n_ (*M,N*) to the overall memory consumption please refer to Kunkel et al. (2014).

Finally, contributions of connections and corresponding infrastructure in the new kernel are described by:

$$\begin{array}{l} M_{\text{c}}(M,\;T,N)\;=K_{\text{M}}^{\text{stat}}\;m_{\text{c}}^{\text{stat}}+K_{\text{M}}^{\text{stdp}}\;m_{\text{c}}^{\text{stdp}}+KN_{M}\;m_{s}\\ \;+\;N_{M}\text{ min}(K,MT)m_{t}. \end{array}$$

The first two components describe the memory consumption of the actual synapse objects, proportional to the local number of synapses of a particular type and the size of an individual synapse ($m_{\text{c}}^{\text{stat}}\text{and }m_{\text{c}}^{\text{stdp}}$ represent the memory usages of a single static or STDP synapse, respectively). The third term is the contribution of the data structure storing the sources of the respective $K_{\text{M}}^{\text{stat}}+K_{\text{M}}^{\text{stdp}}=KN_{M}$ synapses (*m*_s_ is the memory consumption of a single source, see section 3.1.1). The fourth term accounts for the presynaptic part of the two-tier connection infrastructure: each local node needs to store a certain number of targets, each of which consume *m*_t_ bytes (see section 3.1.2). As above, the appearance of min(*K, MT*) is due to adaptations for pre-petascale machines (see section 3.3).

## 3. Results

**Figure 2:**
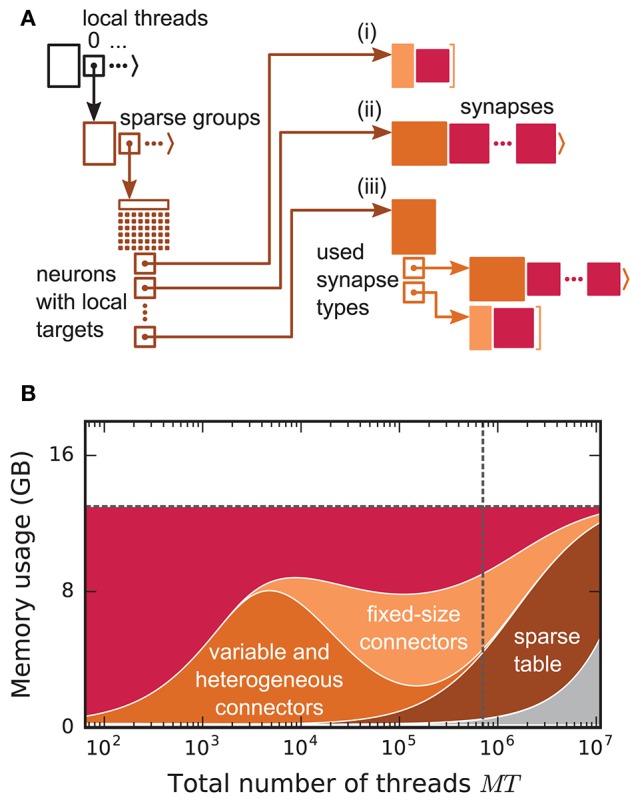
Connection infrastructure and memory usage for state-of-the-art purely postsynaptic storage of connections. **(A)** Connection infrastructure of the previous NEST kernel (4g). Each MPI process maintains a resizable array of pointers (top left; black) to thread-specific sparse tables (bottom left; dark orange), which hold connectivity information for all neurons in the network with local targets and enable efficient access to the corresponding connection objects. Each sparse table consists of sparse groups, where each group is responsible for 48 neurons: One bit per neuron indicates whether the neuron has local targets or not (tiny dark orange squares). If a neuron has local targets, the sparse group stores a pointer to a connector, which holds the local target synapses (pink filled squares). Depending on the number of synapses and the number of synapse types, the connector can hold (i) a small, fixed number of synapses of a single type (top right; light orange), (ii) a variable number of synapses of a single type (middle right; medium orange), or (iii) a connector of type (i) or (ii) for each synapse type that is in use (bottom right; medium orange). **(B)** Estimated memory usage per compute node by different data structures of the 4g kernel as a function of the total number of threads for a maximum-filling scaling with 1 MPI process, 8 threads and 13 GB of main memory per compute node. From top to bottom: synapses (pink), fixed-size connectors [light orange; case (i) in **A**], variable-size connectors and heterogeneous connectors [medium orange; case (ii) and (iii) in **(A)**, respectively], sparse tables (dark orange) and MPI buffers (gray). The dashed horizontal line marks the maximum memory available per compute node; the dashed vertical line marks the maximum number of MPI processes on the K computer.

**Figure 3:**
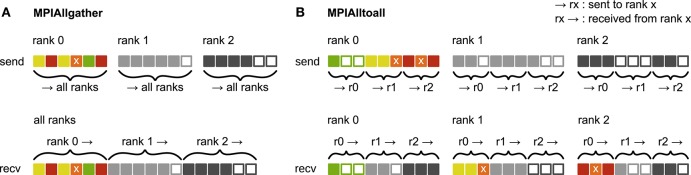
Communication of receiver-selective data using MPI_Allgather and MPI_Alltoall. The panels illustrate send and receive buffers for the example of an MPI communication that involves three ranks. Squares represent single buffer entries. Both collective MPI calls require homogeneous data types and equal send and receive buffer sizes for all ranks, which can entail sending empty buffer entries (unfilled squares). For the data that is sent by rank 0, colors indicate whether the data is required only by rank 0 (green), rank 1 (yellow), rank 2 (red), or both rank 1 and 2 (orange). For clarity, desired destinations for data that is sent by rank 1 and 2 are not indicated. **(A)** MPI_Allgather: All ranks receive the complete send buffer from all ranks, which can include unneeded data (e.g., rank 1 and 2 both receive the required orange entry but they also receive the unnecessary green entry). The receive buffer is a concatenation of all send buffers and the receive buffer size hence scales with the total number of ranks taking part in the communication. **(B)** MPI_Alltoall: Send buffers consist of equally sized sections that are destined for different receiving ranks, which allows each rank to define the data to be transmitted to any particular rank; for example, rank 0 sends the yellow entries only to rank 1. Each rank has to send identically-sized buffer sections to each rank, which can entail sending empty buffer entries or even entirely empty buffer sections. Rank 2, for example, sends an empty buffer section to rank 1. To send specific data to multiple ranks, the sending rank needs to copy the data to the send-buffer sections of all intended receiving ranks, which leads to redundancy in the send buffer; rank 0, for example, sends the orange entry “x” to both, rank 1 and 2. The size of the receive buffers is identical to the size of the send buffers and independent of the number of ranks participating in the communication.

Before presenting the new simulation kernel, we shortly discuss the main bottlenecks of the present technology for large-scale neuronal networks simulations. It was previously suggested that synapse objects should be stored on the compute node on which their target neuron resides (Morrison et al., 2005). On the one hand, this choice reduces the amount of information to be communicated during simulation of the network, while on the other hand it allows for completely parallel network construction, a practical necessity for large-scale simulations. The previous kernel of NEST uses a source-based address-event-representation (AER) scheme (Boahen, 2000; Lansner and Diesmann, 2012), employing MPI_Allgather (see **Figure 3A**, Message Passing Interface Forum, 2009) to communicate spike events among compute nodes: Each MPI process receives the global ids (GIDs) of all neurons that emitted a spike since the last MPI communication took place, and each thread needs to determine the thread-local neurons to which it needs to deliver the events. To this end, each thread is equipped with a sparse table, a memory-efficient hashtable, for efficient lookup of connection objects via the GID of the source (see Figure 2 and Kunkel et al., 2014). This data structure requires few bits per neuron in the network to signal presence or absence of targets for every possible source. For each source with local targets it incurs additional overhead besides the actual connection objects. For a fixed number of connections per neuron and a large number of MPI processes, a neuron typically has none or very few targets on an arbitrarily selected process (Figure 1, cf. Kunkel et al., 2014). Nevertheless, the sparse table occupies a major portion of the available memory in the post-petascale regime (Figure 2, dark orange area, cf. Figure 2 in Kunkel et al., 2014) as its memory consumption grows proportional to the total number of neurons in the network. This overhead limits the scalability of the simulation kernel. In addition, the size of the MPI receive buffers grows proportionally to the total number of MPI processes due to the MPI_Allgather communication scheme. The growth of spike buffers requires not only an increasing amount of memory (Figure 2, gray area) but it also has an impact on simulation speed. For large-scale simulations over thousands of processes, a significant amount of time is spent on skipping spikes in MPI receive buffers from sources that do not have any local target – a contribution to the wall-clock time that grows linearly with the number of MPI processes (Schenck et al., 2014; Kunkel and Schenck, 2017). To better exploit current supercomputers and to ensure scalability of the simulation code beyond the petascale regime, all data structures and runtime contributions that scale either proportional to the number of MPI processes or to the total number of neurons in the network need to be removed: Every process should only store and receive information relevant to local nodes.

Here we propose a solution that employs MPI_Alltoall (see Figure 3B) to communicate spikes in a directed fashion, combined with a two-tier connection infrastructure for spike routing that consists of a presynaptic part, located on the MPI process of the sending neuron and a postsynaptic part, located on the MPI process of the receiving neuron. The connection infrastructure is constructed in two phases: First only the postsynaptic part including the actual connection objects is created and then, prior to simulation, the presynaptic infrastructure is constructed based on the postsynaptic connection information. In the following sections we discuss this new connection infrastructure and its instantiation in detail.

### 3.1. Two-tier connection infrastructure

#### 3.1.1. Postsynaptic infrastructure

**Figure 4:**
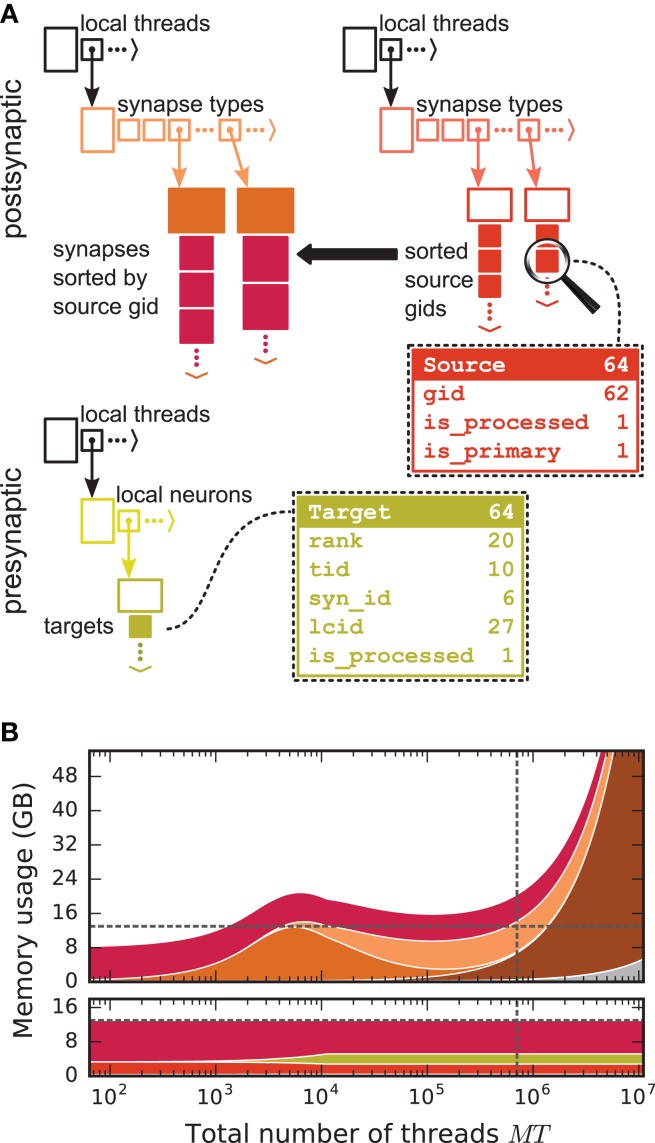
Two-tier connection infrastructure and its memory usage compared to purely postsynaptic storage of connection information. **(A)** Two-tier connection infrastructure of the new NEST kernel (5g). Top: The receiver side of the connection infrastructure consists of two identically structured parts: The connection table (top left) and the source table (top right). Connection table: Each MPI process maintains a resizable array of pointers (black) to thread-specific resizable arrays of pointers to variable-sized containers for every synapse type. If a synapse type is in use, the corresponding container (orange filled rectangle) stores all thread-local synapses of this type (pink filled squares) in a resizable array. Synapse types can differ in memory consumption per object, indicated by different sizes. In each container, synapse objects are sorted by GIDs of the presynaptic neurons. Source table: Source objects (red filled squares) are stored in a three-dimensional resizable array, with a one-to-one relation between each source object and the synapse object in the same position in the connection table. Sources contain the GIDs of the presynaptic neurons and two markers (Source bit fields shown in dashed-line rectangle). Sender side of the connection infrastructure (bottom): Each MPI process maintains a target table, which is a resizable array of pointers (black) to thread-specific resizable array of resizable arrays that store the Target objects (green filled squares) for every thread-local neuron. The Target objects contain the locations of the targets, in terms of the MPI ranks and the three-tuple (tid, syn_id, lcid) that identifies the synapses on the target side in the corresponding connection table (Target bit fields shown in dashed line rectangle). **(B)** Estimated memory usage per compute node by different data structures of the 4g (top) and 5g (bottom) kernel as a function of the total number of threads for a maximum-filling scaling of the 5g kernel with 1 MPI process, 8 threads and 13 GB of main memory per compute node. Color code for the 4g kernel as in Figure 2. Color code for the 5g kernel from top to bottom: synapses (pink), targets (green) and sources (red). Memory usage of MPI buffers is not visible due to the small buffer size. The dashed horizontal line marks the maximum memory available per compute node; the dashed vertical line marks the maximum number of MPI processes on the K computer.

We first focus on the postsynaptic part of the connection infrastructure. On each process, we maintain two identically structured three dimensional resizable arrays implemented using the vector container of the C++ Standard Template Library, the first storing connections that have process-local targets and the second their corresponding sources (Figure 4A, top). Upon creation of a connection, the actual connection object and the source GID are pushed-back into the innermost dimension of these resizable arrays indexed by the thread of the target neuron (first dimension) and type of the connection (“synapse type,” second dimension). A single connection object contains all parameters and status variables of a synapse as well as a function to obtain a pointer to the target neuron. In these three dimensional structures, a three-tuple consisting of three integers describing local thread id, synapse id and local connection id, uniquely identifies a connection object on a specific MPI rank and can hence be used in a target-based AER scheme. In contrast to the previous generation kernel, connections are no longer separated according to source GID (cf. Figure 2), thus avoiding overhead per potential source. The size of the data structure is therefore independent of the total number of neurons in the network. The removal of the separation by source also allows us to allocate the majority of the memory required to store all connection objects as a single chunk. To improve the size to capacity ratio of the used resizable array, we use a resizing strategy that does not double the capacity each time the maximal capacity is reached, but allow the growth factor to be adjusted at compile time, with a default that increases the size of the resizable array by 50 %. The additional storage of the GIDs of all source neurons in the second structure is required for the construction of the presynaptic part of the connection infrastructure (see section 3.1.3). This information also allows the user to query connectivity without requiring extensive MPI communication among all processes. Two additional flags are required within the source objects for construction of the presynaptic part of the connection infrastructure. They are implemented as bit fields to reduce the memory footprint (Figure 4).

#### 3.1.2. Presynaptic infrastructure

We now turn to the presynaptic part of the connection infrastructure required to implement directed communication of spikes. To determine the target processes to which a spike needs to be delivered, we need information about the locations of all postsynaptic targets on the process of the sending neuron. The presynaptic infrastructure consists of a single three dimensional resizable array which stores the location of all targets from outgoing connections of local neurons (Figure 4A, bottom). The global location of a connection is uniquely given by the rank on which the target neuron is located in combination with the three-tuple for locating the connection in the postsynaptic structure on the target rank, as described in the previous section. To reduce the memory usage of this additional part of connection infrastructure, we have combined this location information in each object via bitmasks to fit into 8 B, which strictly limits the maximal values of the individual fields (cf. Figure 4A, bottom). The objects containing the location of all targets are stored according to the thread of the source (first dimension) and the thread-local id of the source (second dimension).

#### 3.1.3. Construction of presynaptic connection infrastructure

**Figure 5:**
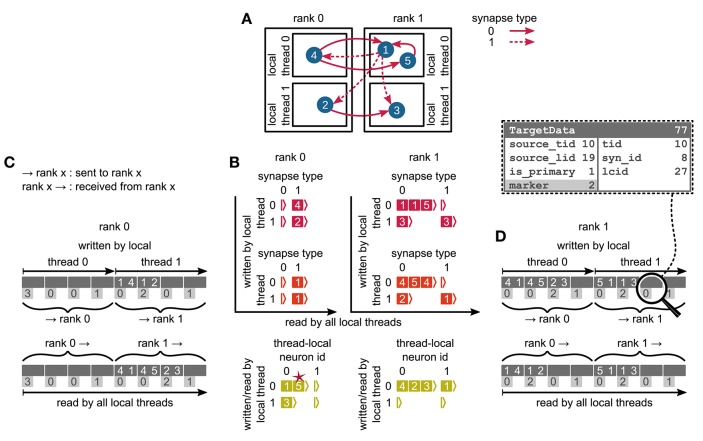
Communication of connectivity data from postsynaptic to presynaptic side for the two-tier connection infrastructure. Example network of 5 neurons **(A)** with global identifiers (GIDs) 1 to 5 (blue filled circles with white numbers) that are connected via two different types of synapses (pink arrows); for simplicity, the two types have synapse-type index 0 and 1 (solid and dashed arrows, respectively). Neurons are distributed across 2 MPI processes (outer rectangles) and 2 threads per process (inner rectangles); 4 threads in total. Synapses are hosted by the threads of their postsynaptic neurons. **(B)** From top to bottom: Connection table, source table, and target table of the example network in **(A)** on rank 0 (left) and rank 1 (right). Color code as in Figure 4A: Synapses, sources, and targets shown as pink, red, and green filled squares, respectively, where white numbers indicate target GIDs, source GIDs, and target GIDs again, respectively. The pink star indicates redundant connection information that is absent in the optimization for small-scale simulations (cf. section 3.3). All tables are three-dimensional resizable arrays: Outermost resizable arrays for threads (vertical axes), middle resizable arrays for synapse types or local neurons (horizontal axes), innermost resizable arrays that hold the individual objects indicated by chevrons. When two neurons are connected, the thread of the postsynaptic neuron adds the new synapse to the connection table and a corresponding Source entry to the source table. Connectivity data needs to be communicated to the presynaptic side at the beginning of the simulation in order to construct the target table. **(C,D)** MPI send buffer (top) and receive buffer (bottom) that contain the TargetData of the example network, for rank 0 and rank 1, respectively; TargetData bit field shown in dashed line rectangle. Top rows (dark gray): Each field contains zero or two entries, which indicate the (source GID, target GID)-tuple. Bottom rows (light gray): Flags in each TargetData used for communication of status values among all processes (0: default, 1: no more data to send, 2: end of valid data in section, 3: skip this section).

We split network construction into two phases. The first phase stores connections and source GIDs in the postsynaptic connection infrastructure on the processes of the corresponding target neurons. The second phase, initiated by the simulation kernel just before the simulation starts, constructs the presynaptic infrastructure on all processes (see Figure 5 for an example). All processes containing neurons with outgoing connections require information about the location of the corresponding targets. This information is communicated using MPI_Alltoall such that each rank receives only data about targets of its process-local neurons. While constructing the MPI send buffers on the postsynaptic side, each local thread is responsible for gathering the relevant information from the postsynaptic data structures for a consecutive range of ⌈*M*/*T*⌉ source ranks. This design choice removes the strict separation of data structures by threads used in previous kernel versions. To construct the send buffer, each thread reads through all sources, determines the source rank for each connection based on the source GID and the round-robin distribution of neurons, and, if the source rank falls in the thread's assigned interval of ranks, creates an entry in the corresponding part of the MPI buffer (see Figure 3B for an illustration of how different parts of the MPI buffers are distributed across ranks by MPI_Alltoall). A single entry in the MPI buffers comprises the thread and local id of the source neuron, the rank of the target neuron and the three-tuple identifying the corresponding connection in the postsynaptic structure. After the buffers have been exchanged via MPI_Alltoall, all threads read through the MPI receive buffers, each considering only entries relevant for thread-local neurons. From the relevant entries, the rank of the target neuron and the three-tuple are inserted into the presynaptic infrastructure. Prior to filling the send buffer, we use a collective MPI call to determine the maximal number of buffer entries that need to be communicated between any two ranks. This number is used to determine the required size of the MPI buffers in order to communicate all connection information in a single MPI call. The maximal size of the MPI buffers can be limited to avoid temporary peaks in memory consumption. In case this size is smaller than the required size, additional communication rounds are initiated until all connection information is communicated among all ranks. The current maximal MPI buffer size for communicating connection information is 128 MB, which balances memory usage and number of communication rounds for our benchmark model. Under different circumstances, users might, however, want to reduce the number of communication rounds at the expense of larger memory usage or vice versa.

### 3.2. Communication of spikes

**Figure 6:**
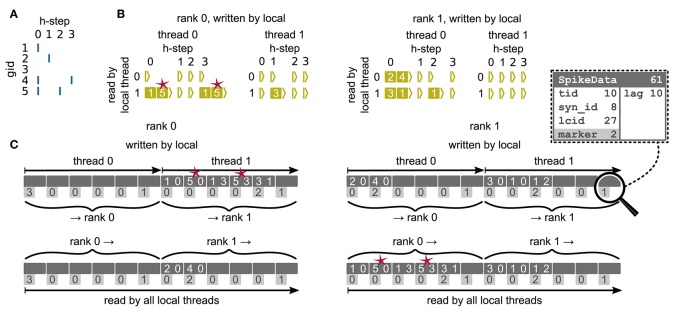
Communication of spike data using MPI_Alltoall for the example network in Figure 5A and an example activity **(A)**, where a communication step consists of four neuronal update steps (*h*-steps); spikes are shown as blue bars. **(B)** Spike register, which temporarily buffers spikes before they are collocated in the communication buffers on rank 0 (left) and rank 1 (right). Numbers indicate target GIDs. Pink stars indicate redundant information, absent in the optimization for small-scale simulations (cf. section 3.3). **(C)** MPI send buffers (top) and receive buffers (bottom) for rank 0 (left) and rank 1 (right) that contain SpikeData for the example activity. SpikeData bit fields shown in dashed rectangle. Top rows: Each field contains zero or two entries, which indicate the (target GID, lag)-tuple. Bottom rows: Flags in each SpikeData used for communication of status values among all processes (0: default, 1: no more data to send, 2: end of valid data in section, 3: skip this section).

We now discuss the communication of spikes from the presynaptic to the postsynaptic ranks, making use of the two-tier connection infrastructure introduced above. Morrison et al. (2005) observe that the dynamics of neurons are decoupled from each other for the period of the minimal delay in the network. The authors exploit this insight by introducing a communication scheme that uses collective MPI calls for exchanging spikes at regular intervals determined by the minimal delay, instead of the typically much smaller simulation resolution. Here we rely on the same causality constraints to disentangle communication interval and simulation resolution, but modify the communication algorithm to make use of directed communication via MPI_Alltoall. During a communication interval, spikes are buffered in a four dimensional resizable array (Figure 6) and only transferred to the MPI send buffers at the end of the interval, right before the MPI communication takes place. In the four dimensional data structure, the first dimension corresponds to the thread of the source neuron, the second dimension corresponds to the thread responsible for writing the respective entries to the MPI buffer (see below), and the third dimension corresponds to the relative simulation step within the current communication interval at which the spike occurred (“lag”). In a similar way as in the collocation of the send buffer during communication of connection information, each thread is assigned a range of receive ranks for which it is responsible, corresponding to particular sections in the MPI send buffer (see Figure 3, cf. section 3.1.3). By splitting the MPI send buffer in this fashion, buffers can be collocated in parallel, without requiring additional checks to avoid that threads are writing to the same memory address. When a neuron spikes, the corresponding thread retrieves the locations of all its outgoing connections from the presynaptic infrastructure (section 3.1.2). For each entry the thread appends a copy of each target at a specific position in the four dimensional spike buffer, determined by the thread of the source neuron (first dimension), the id of the thread that according to the rank of the target neuron will later create the corresponding entry in the MPI buffer (second dimension), and the current lag (fourth dimension). The second dimension is introduced in order to read the spike buffer in parallel at the end of the communication interval: Each thread only processes its (private) share of the spike buffer and creates entries in the MPI buffer accordingly. An entry comprises the three-tuple identifying a connection on the receiving rank through which the spike should be delivered to the target neuron, while the receiving rank is implicitly encoded by the entry's position in the MPI send buffer. After the spike buffer has been completely processed or in the case that only spikes are left that cannot anymore be accommodated in the MPI buffer, buffers are exchanged among all compute nodes via MPI_Alltoall. Directly after the MPI communication, all threads read the process-local MPI receive buffers and deliver spikes only via thread-local connections. Several of such communication rounds may be required until all MPI processes have completely exhausted their spike buffers. Each additional communication round at the end of a single communication interval increases the size of the MPI buffer up to a certain, user adjustable, maximal size. This dynamic resizing allows the MPI buffers to maintain the minimal required size, reducing the total number of required communication rounds. After all ranks have processed their spike buffers, the simulation resumes with updating the nodes for the next interval.

### 3.3. Adaptations for the pre-petascale regime

The two-tier infrastructure introduced above is designed for the regime where each neuron has either no or only few targets on each thread (*K* ≪ *MT*; “sparse limit,” Kunkel et al., 2014), motivated by the sub-optimal memory usage of the previous technology in this limit (Figure 2). However, in the pre-petascale regime, neurons typically have many targets per thread (*K* ≫ *MT*, see Figure 1). This results in performance degradation of the new simulation technology compared to the previous technology, due to: (i) reduced memory locality in the postsynaptic infrastructure as connections are not separated and stored sequentially by source, leading to hardly predictable random memory access during spike delivery and (ii) redundancy in communication of spikes as each source sends an individual spike to each of its targets and hence possibly multiple spikes to each thread (red stars in Figure 6). In the extreme case of a single MPI process with a single thread, each neuron generates *K* spikes prior to MPI communication, instead of just a single packet that is multiplied during delivery on the postsynaptic side as it was the case for the previous technology. On these grounds Morrison et al. (2005) argue that for a small number of processes, a sender-based AER scheme is most efficient.

Here we solve both shortcomings by sorting connections in the postsynaptic infrastructure by their corresponding source GIDs (Figure 4). A spike delivered to the first connection originating from a specific source can then (locally) be passed to all subsequent connections of the same source, leading to linear memory access patterns. The source neuron hence only needs to send a single spike to each thread on which it has targets, postponing some of the spike duplication to the postsynaptic delivery. In addition to solving the above issues, this reduces the memory usage of the presynaptic infrastructure in the regime where neurons have multiple targets per thread, as a single neuron only needs to store the location of *MT* targets instead of *K* targets. During construction of the presynaptic connection infrastructure each thread only communicates the location of the first connection for each combination of source and synapse type. Due to the organization of the data structure, sources can only be sorted per synapse type. Therefore, this optimization is only effective if a minimal number of different synapse types necessary for a particular simulation is used. For example, in the NEST simulation software the CopyModel mechanism is a convenient way to create a synapse type that differs from the original by the values of default parameters. This makes the description of the network model in the simulation language more compact and readable. The consequence, however, is that the above optimization does not work as efficiently as for a model description where varying synapse parameters are explicitly set for different synapse instances of the same type. As the number of MPI processes increases the benefit of sorting connections by source diminishes. Not using the optimization saves time during network construction for large-scale simulations by avoiding the then superfluous sorting step (section 3.5).

### 3.4. Memory usage of the new simulation kernel

The memory-usage model introduced in section 2.5 exposes the differences between the previous (4g) and the new (5g) simulation kernel in total memory usage per MPI process of our reference network (section 2.1). Here we consider a maximum-filling scenario (section 2.2) for the 5g kernel using 13 GB per MPI process. In the previous kernel, the sparse tables and MPI buffers consume memory proportionally to the total number of neurons in the network ultimately limiting the scalability of the simulation code (Figure 2). Removing the sparse table and the constant overhead for all sources with local targets by introducing a two-tier connection infrastructure significantly reduces the memory usage in the petascale regime and beyond (*MT* > 10^5^, see Figure 4). Additionally, replacing MPI_Allgather with MPI_Alltoall reduces the size of MPI receive buffers by eliminating their growth with the total number of processes. These changes result in perfect scaling of the postsynaptic data structures with respect to memory usage. The new technology allows for simulations of larger networks than the previous kernel using the same computational resources in the regime of more than a few hundred MPI processes (Figure 4). For more than 10^4^ threads, also the presynaptic infrastructure consumes constant memory as its size is proportional to the average out-degree of the neurons (~*K*). In the regime up to 10^4^ threads, we achieve decreased memory usage of the presynaptic infrastructure due to the optimizations described in section 3.3. In this regime, each neuron typically has many targets on each thread, such that the size of the presynaptic infrastructure is proportional to the total number of threads (~*MT*). Below approximately 10^3^ threads, the previous technology consumes less memory as it does not require storage of the source GID for every connection. In addition, the size of STDP synapse objects has increased, since each STDP synapse now needs to store the time of the last spike of its source, which previously only needed be stored once per source neuron on each thread. To reduce the total memory consumption, the stored source GIDs can optionally be removed while constructing the presynaptic data structures. This however implies that after the presynaptic infrastructure has been created, network connectivity can neither be changed nor queried any more.

### 3.5. Performance of new simulation kernel

To investigate the performance of the new technology we next carry out scaling experiments using the archetypal network model outlined in section 2.1. In the following, we use the terms “build time,” “init time,” and “sim time” to refer to the measured wall-clock times of three successive phases of the benchmark-network simulations. The build time is the wall-clock time required to construct all nodes and the postsynaptic part of the connection infrastructure. After the build phase a very short simulation of 10 ms biological time is followed by the actual simulation of 1 s biological time; the measured wall-clock times are referred to as init time and sim time, respectively. The init time accounts for the construction of the presynaptic part of the connection infrastructure in 5g (see section 3.1.3), but also for the initial resizing of MPI buffers in both 4g and 5g. Besides, initial transients of the network dynamics subside during the pre-simulation. In principle, these two contributions could be measured separately, but since the largest contribution arises from the construction of the presynaptic part of the connection infrastructure, we do not separate these any further. In the following we refer to the 5g kernel version that uses the adaptations for the pre-petascale regime described in section 3.3 as “5g-sort” and to the version without these adaptations as “5g-nosort”; collectively we refer to both versions as “5g.”

#### 3.5.1. Weak scaling

In weak scaling benchmarks, the problem size per compute node, or equivalently per MPI process, is fixed while the total number of compute nodes increases (section 2.2). In this scenario a perfectly scalable simulation kernel shows constant run time for all phases and constant memory usage per compute node. We simulate our benchmark-network model (section 2.1) on JUQUEEN with *NM* = 18,000 neurons per compute node and *K* = 11,250 incoming synapses per neuron while build time, init time, sim time, and memory usage per compute node assess the performance of the 4g, 5g-sort, and 5g-nosort variants.

**Figure 7:**
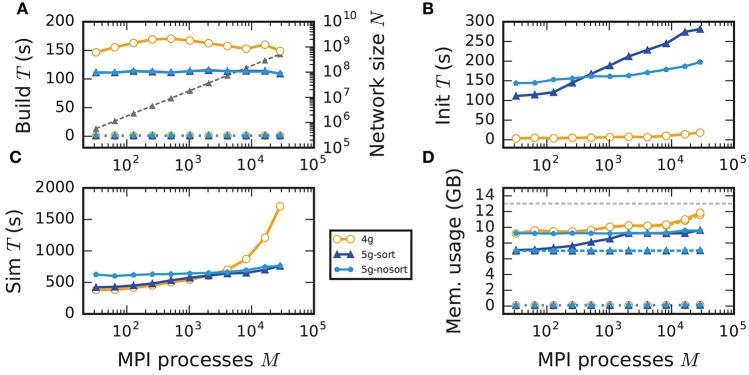
Weak scaling of an archetypal neuronal network simulation on a petascale computer. Runtime and memory usage per compute node for an increasing number of MPI processes *M* ∈ {32; 64; 128; 256; 512; *1024*; *2048*; *4096*; *8192*; *16, 384*; *28, 672*} with one MPI process and *T* = 8 threads per compute node on JUQUEEN. Each compute node hosts *NM* = 18,000 neurons with *K* = 11,250 incoming synapses per neuron. Network dynamics simulated for 1 s of biological real time. Color code in all panels: 4g (orange), 5g without optimizations for pre-petascale (5g-nosort, light blue), and 5g with optimizations (5g-sort, dark blue). **(A)** Build time of nodes (dashed lines) and connections (solid lines); 5g-sort data covered by 5g-nosort data. Gray triangles and dashed line show the total network size *N* (right vertical axis). **(B)** Init time. **(C)** Sim time. 4g data partly covered by 5g-sort data. 5g-sort data partly covered by 5g-nosort data. **(D)** Cumulative memory usage for a single MPI process after construction of neurons (dotted lines; < 140 MB), after construction of connections (dashed lines), and after simulation including pre-simulation (solid lines). 5g-sort data partly covered by 5g-nosort data. Horizontal gray dotted line indicates the maximum memory available on a single compute node of JUQUEEN.

The build time shows almost perfect scaling for both 4g and 5g, as construction of the postsynaptic part of the connection data structures is completely parallel and does not involve any communication among compute nodes (Figure 7A). While the build time for nodes is negligible for both technologies, construction of connections is about 25–30 % faster in the new kernel. This is due to a more efficient memory allocation for synapse objects in 5g: The major part of the memory that is required to store all local connections of a specific type is allocated en bloc (section 3.1.1). Ippen et al. (2017) discuss the intricacies of memory allocation during network construction in the previous kernel. Note that for simulations using the previous kernel, we preload jemalloc (Evans, 2011) to replace the default allocator. Jemalloc was specifically designed to improve allocation for multi-threaded applications and significantly reduces the build time for the previous simulation kernel in multi-threaded environments compared to the default allocator (Ippen et al., 2017).

**Figure 8:**
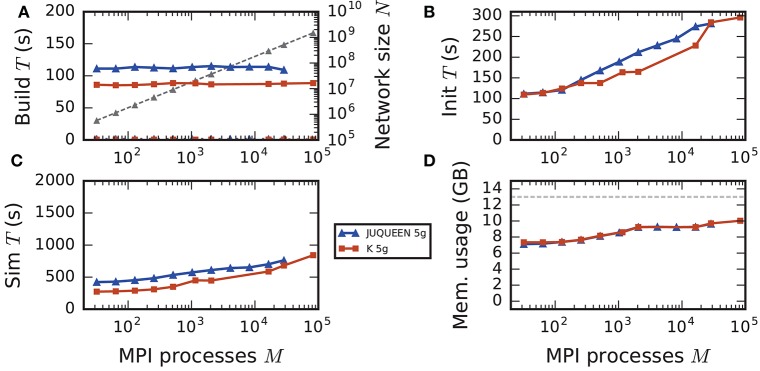
Comparison of weak scaling on two petascale systems, JUQUEEN and K computer. Color code in all panels: 5g-sort on JUQUEEN (dark blue), 5g-sort on the K computer (dark red). See Figure 7 for details. Number of MPI processes: *M* ∈ {32; 64; 128; 256; 512; 1152; 2048; 16,384; 28,672; 82,944} with a single MPI process per compute node and *T* = 8 threads per process.

In the following discussion we focus on the comparison of 4g and 5g-sort, and afterwards consider the differences to 5g-nosort. After construction of the postsynaptic data structures in the new kernel, the connection information needs to be communicated to the presynaptic side, leading to a significant increase in init time compared to the previous kernel (Figure 7B). For a small number of MPI processes (≲ 128) the init time is evenly spent on sorting connections by the respective sources and on collocating connection information to the MPI send buffers. To collocate the MPI buffers, all threads in a single process need to read through the whole postsynaptic data structures and therefore the performance gain from multiple threads is small in this phase. For a larger number of processes the contribution from collocating buffers increases significantly over the investigated range, dominating the init time in the regime of 10^4^ MPI processes and leading to a significant increase in init time over the investigated range of MPI processes (Figure 7B). On the one hand, the increase of work load is due to the growing amount of connectivity data that needs to be exchanged between processes, because targets of individual neurons are distributed over more and more ranks (cf. section 3.3). On the other hand, the increase is due to suboptimal usage of the available space in the MPI buffers caused by the random out-degree of nodes. The init time, however, is a constant contribution to the total wall-clock time and therefore its relative impact decreases for longer simulation times. The sim time is approximately equal in the two kernel versions up to about 2048 compute nodes (Figure 7C). For larger numbers of MPI processes, the sim time in 4g increases approximately linearly with the number of MPI processes due to the linearly increasing size of the MPI receive buffers. Since the receive buffer size stays approximately constant in 5g, sim time increases only slightly, leading to much better scaling behavior and a decrease in sim time by more than 55 % for full JUQUEEN simulations. The main contribution to the sim time are the delivery of spikes from the MPI buffers via the respective connections to the target neurons, including weight updates of plastic synapses, followed by the time spent on propagating the neuronal dynamics. Over the investigated range of MPI processes, communication time takes up only a small part of the total simulation time (**Figure 12**). The relative contribution of communication, however, depends on the minimal delay in the network, which determines the communication interval (here *d*_min_ = 1.5 ms). Communication can become a relevant factor in simulations with small delays on the same order of magnitude as the simulation resolution (*d*_min_~0.1 ms). The memory usage per compute node is consistently smaller in the new kernel than with the previous technology (Figure 7D); in particular it is approximately constant above ~2048 processes as predicted by the memory-usage model (section 3.4), demonstrating its perfect scaling behavior. In this regime, most memory is allocated for the individual connection objects, followed by contributions from the postsynaptic infrastructure responsible for storing the source of each connection and a significant contribution from the presynaptic infrastructure storing the targets of neurons. The slight increase in memory usage for more than 10^4^ processes is most likely caused by an increased memory usage of the MPI library. For a smaller number of MPI processes, the optimizations for small-scale simulations reduce the size of the presynaptic data structures significantly and hence the memory usage of the new kernel by about 20 % compared to the case without optimizations (Figure 7, blue vs. light-blue). In addition to simulations on JUQUEEN, we perform weak-scaling benchmarks on the K computer. The results are very similar to the data obtained from JUQUEEN (Figure 8), demonstrating the excellent scalability of the simulation kernel to almost 10^5^ MPI processes. The increase in memory consumption between 16,384 and 82,944 MPI processes (Figure 8D) can be traced back to the increased memory usage of the MPI library.

Without optimizations for small-scale simulations (5g-nosort), the init time increases for a small number of MPI processes (*M* < 10^3^) compared to 5g-sort due to an increased amount of connection information that needs to be communicated among ranks. However, as the number of MPI processes increases, a single neuron has a decreasing number of targets on each process, diminishing the advantage of sorting connections by source. In this regime, we save time by not sorting connections without incurring a penalty, leading to 5g-nosort having a smaller init time than 5g-sort from about 1024 MPI processes on. Also the sim time for 5g-nosort is increased in the regime of few MPI processes, due to a larger amount of spikes that need to be communicated: one for each target per thread, instead of just one for the first target per thread. This disadvantage diminishes with increasing number of MPI processes, and 5g-sort and 5g-nosort exhibit similar sim times from about 2048 MPI processes on. In contrast to 5g-sort, memory usage for 5g-nosort is constant since the size of the presynaptic data structures are independent of the number of MPI processes also for *MT* < *K*.

#### 3.5.2. Strong scaling

**Figure 9:**
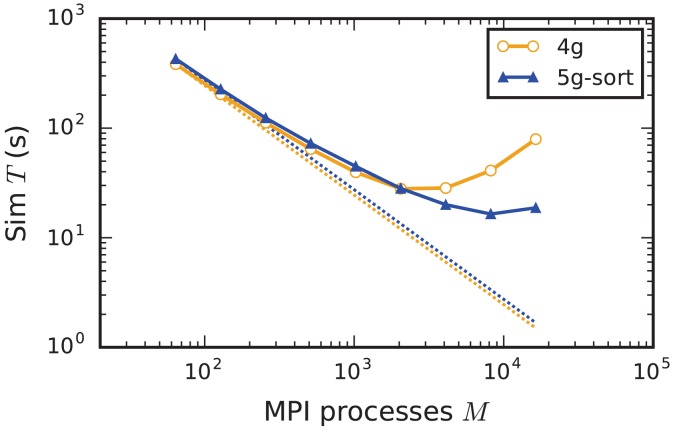
Strong scaling of simulation time of an archetypal neuronal network simulation on a petascale computer. Runtime for an increasing number of MPI processes *M* ∈ {64; 128; 256; 512; 1024; 2048; 4096; 8192; 16,384} and *T* = 8 threads per process on JUQUEEN. Same color code, marker styles, and line styles as in Figure 7. Straight dotted lines indicate perfect scaling. *N* = 1, 152, 000.

Increasing the number of MPI processes in a strong scaling scenario (section 2.2) shows perfect scaling of the build time for both the old and the new simulation kernel since construction of nodes and the postsynaptic part of the connection infrastructure are entirely parallel and do not require any communication (see Figure A5 in the Appendix). Also the init time shows almost perfect strong scaling for the new kernel. For the previous kernel, the sim time initially scales very well, but saturates between 2048 and 4096 MPI processes; it even increases for larger numbers (Figure 9), most likely due to the increasing size of MPI receive buffers as reading of the buffers introduces significant overhead during spike delivery. Using the new kernel, the sim time scales well up to 8192 processes where it saturates, simulating 1 s of biological real time in 16 s of wall-clock time for a network with more than 10^6^ neurons and almost 10^10^ plastic synapses (Figure 9). Deviations from perfect scaling can mainly be traced back to MPI communication, which puts a strict lower bound on potential optimization of the simulation time. The memory consumption of the new kernel before construction of the presynaptic infrastructure scales perfectly with increasing number of MPI processes. However, as targets of neurons become distributed across more and more MPI processes, the size of the presynaptic data structures increases, leading to slightly higher memory usage for a large number of MPI processes (Figure A5).

#### 3.5.3. Maximum-filling scaling

**Figure 10:**
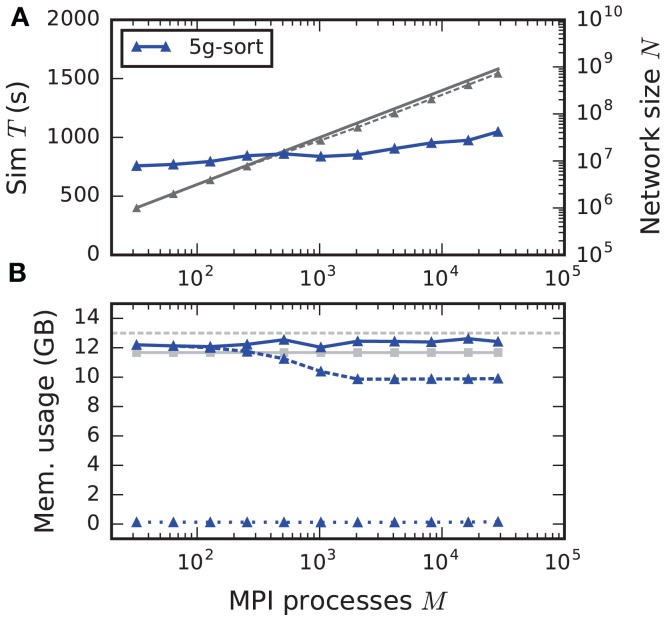
Maximum-filling scaling of an archetypal neuronal network simulation on a petascale computer. **(A)** Runtime and **(B)** memory usage per compute node for an increasing number of MPI processes *M* ∈ {32; 64; 128; 256; 512; 1024; 2048; 4096; 8192; 16,384; 28,672} with one MPI process and *T* = 8 threads per compute node of JUQUEEN. Same color code, marker styles, and line styles as in Figure 7. Gray solid line in **(A)** indicates network size for perfect scaling. Light gray solid line with square markers in **(B)** is the prediction of the memory-usage model.

The maximum-filling scaling scenario keeps the memory usage per compute node approximately constant and close to the maximal memory available. This exposes the limits of the new technology in terms of the maximal network size on a given hardware (section 2.2). Since without optimizations the memory usage per compute node stays constant, weak scaling and maximum-filling scaling are identical in that case. Here we consider the case with optimizations (5g-sort). The memory usage of the presynaptic connection infrastructure is negligible for small-scale simulations and increases as the targets of each neuron become more and more distributed across different compute nodes. The number of neurons that fit on a single compute node consequently decreases, up to about 2048 processes (Figure 10). For a larger number of MPI processes, the number of neurons fitting on one compute node stays constant, allowing us to simulate about 730 million neurons connected with about 8 trillion synapses employing the entire JUQUEEN supercomputer. Similar to the weak scaling scenario, simulation time scales well across the investigated number of processes, increasing by less than 45 % for an almost 1000-fold increase in network size. The memory-usage model delivers a fairly accurate prediction of the actual memory usage of a simulation (Figure 10, compare light gray and dark blue solid lines) with a deviation of about 5 %. This mismatch is most likely caused by dynamically resizing containers in the connection data structures for which an accurate estimate of the actual memory usage is difficult.

#### 3.5.4. Performance for small-scale simulations

**Figure 11:**
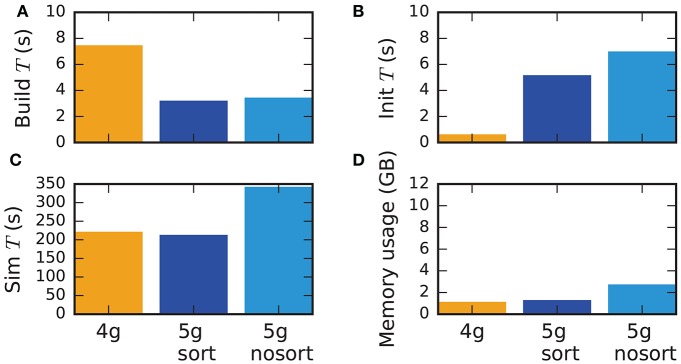
Performance of small-scale simulations. Comparison of **(A)** build time, **(B)** init time, **(C)** sim time and **(D)** memory usage for a down-scaled typical network model on a laptop for 4g (orange), 5g-sort (dark blue) and 5g-nosort (light blue). *N* = 11, 255 and *K* = 3750. Simulations run for 1 s of biological real time.

Although the data structures and algorithms presented in this work are designed to improve the scalability of simulations on large-scale HPC systems, optimizations for small-scale to medium-scale systems (section 3.3) assure that the proposed solution does not impair the performance on laptops, workstations, and small clusters. A down-scaled version of the network model used throughout this study (section 2.1) assesses the performance of the new technology in light of the old (Figure 11). The test system is a Lenovo X250 equipped with an Intel Core i7-5600U processor running at 2.6 GHz with 8 GB of main memory. Simulations are performed using two MPI processes with two threads each. Similar to the results on HPC systems, the build time of nodes and postsynaptic connection infrastructure decreases by 50 % compared to the previous technology (Figure 11). As before, the init phase is, however, much longer due to the required construction of the presynaptic part of the connection infrastructure. In total this leads to comparable network construction time for the previous and new technology (build time + init time). The sim time is almost identical in 4g and 5g. This time is almost exclusively spent on delivering spikes to neurons and on updating plastic connections, both of which are of similar computational complexity in the two technologies. Without optimizations for small-scale simulations, the sim time increases by about 75 %, explicitly demonstrating the necessity of these modifications. Memory usage is also almost identical for the two technologies in the presence of the optimizations. Otherwise, the presynaptic part of the connection infrastructure consumes a significant amount of additional memory.

## 4. Discussion

Well-tested high-performance simulation tools are a prerequisite for advancing neuroscientific modeling of large-scale networks at cellular resolution. The complex connectivity of biological neuronal networks poses a challenge for the numerical simulation of such systems. Previous technologies scale neuronal network simulations to the largest computing systems available today. The work that we present here explores the scalability of new data structures and communication schemes in terms of memory usage and runtime on post-petascale systems and proposes concrete implementations.

The investigation starts by identifying scalability bottlenecks in the previous simulation kernel (4g) of the NEST simulator: In large-scale simulations, a prohibitively large fraction of the available memory on each compute node is taken up by the connection infrastructure and MPI buffers. Therefore, we subsequently design scalable alternative data structures that fully exploit the sparsity of large-scale networks distributed over tens of thousands of MPI processes. The goal of these technological improvements is not to reduce memory consumption *per se*, but to decouple memory consumption per MPI process from the total network size and the total number of MPI processes. In the new kernel (5g) we introduce a two-tier connection infrastructure that combines a postsynaptic part located on the same rank as the target neurons with additional presynaptic data structures located on the ranks of the sending neurons. The presynaptic structure allows us to introduce directed MPI communication via MPI_Alltoall and to remove non-essential information from the postsynaptic rank. This design choice significantly improves the scalability of the simulation kernel without sacrificing flexibility and generality. Through the introduction of the two-tier connection infrastructure and the direct communication via MPI_Alltoall, the new kernel achieves perfect scaling in terms of memory consumption, which prepares the simulation technology for next generations of large-scale HPC facilities. The new data structures and communication schemes enable the simulation of a network of 1.51·10^9^ neurons and 16.8·10^12^ synapses on the K computer (Figure 8), in terms of connectivity slightly larger than the previously reported (Kunkel et al., 2014) simulation with 11.1·10^12^ synapses. The simulation using the new kernel does not require the full main memory available on the K computer; the maximal network size could be increased by about 20 % before running out of memory.

Our work focuses on a decrease in memory usage for post-petascale systems – yet the new data structures and communication framework lead to significant performance gains for large-scale simulations on current supercomputers. Simulations employing the full supercomputer JUQUEEN now run up to 55 % faster than previously, mainly due to the fixed size of MPI receive buffers. The novel technology profits from sending only the relevant spikes to each process: For more than 10^4^ MPI processes, most of the spikes arriving via MPI_Allgather in the old scheme are not required locally, but still need to be processed, leading to a large serial overhead during spike delivery (Schenck et al., 2014; Kunkel and Schenck, 2017). The constant size of the MPI receive buffer, independent of the number of MPI processes, also significantly improves the strong scaling behavior. For 1.1·10^6^ neurons, previous simulation code shows a reduction in wall-clock time only up to 2048 processes, after which it increases again. Employing the new technology, simulation speed increases up to 8192 processes, enabling the simulation of more than one million neurons with plastic synapses for 1 s biological time in 16 s wall-clock time. Although the design of the new technology focuses on the regime beyond 10^4^ MPI processes, optimizations for small-scale simulations on laptops and workstations make sure that the alterations have no negative impact on the overall performance. While without these, performance of the new simulation kernel is reduced for simulations involving less than about 2000 MPI processes, including optimizations leads to almost identical performance of the previous and new simulation kernel even in laptop-scale simulations. The additional time spent on the init time in the new kernel is compensated for by a reduced build time. While this study considers the concrete use case of the NEST simulator in order to obtain quantitative data, the concepts that are presented here are of general nature: The new data structures and algorithms can be implemented in any neural simulator that abstracts neurons and synapses as individual objects allocated as a whole on a single compute node.

The new simulation kernel also mitigates memory allocation bottlenecks observed in the previous kernel when using non-thread-aware allocators (Ippen et al., 2017). The previous technology stores connections in dynamic containers separated according to source neurons, each container storing very few entries in the case of large networks (Kunkel et al., 2014). Due to this separation many small objects were allocated during network construction. In the extreme case of a full JUQUEEN simulation this means that each of the approximately 10^8^ synapse objects per compute node plus its container are allocated separately. In the new technology, all synapse objects of the same type on the same thread are stored in a single container irrespective of their source neurons. Connection routines can thus predict the total required memory and allocate it en bloc prior to instantiating the individual connection objects, which leads to a reduced build time, even with default allocators. However, not all connection builders currently available in simulation codes like NEST support this prediction and it needs to be investigated for which connection routines such a strategy is viable. The removed separation according to source neuron also allows us to keep the new data structures simple, relying only on resizable arrays, without the need for custom container types. The memory-efficient containers developed for the previous kernel might nevertheless become relevant in particular scenarios not investigated in this manuscript.

For large-scale simulations employing thousands of MPI processes, the previous technology does not exhibit good scaling over threads (Figure A4). The main reason for this is the scanning of the large MPI receive buffers, which need to be read completely by all threads. The buffers in the new technology are independent of the number of MPI processes and for a large number of MPI processes hence much smaller than previously. The reduced buffer size not only reduces the absolute time spent in this serial section of the code but also increases the fraction of time spent in fully parallelized sections. Therefore, the new kernel better exploits the computational power of JUQUEEN when running 64 threads per compute node than the previous kernel, which shows negligible speed-up from 8 to 16 threads in full JUQUEEN simulations with 28,672 MPI processes (Figure A4). After speed-up diminishes, 4g also reaches another limit on JUQUEEN: From 32 threads on there is not enough memory available to represent the required data structures. When running 8 threads, 5g reduces the wall-clock time required for the propagation of network dynamics by about 55 % for a full JUQUEEN simulation compared to 4g. By increasing the number of threads to 64, which is the maximal number of threads per compute node on JUQUEEN, we achieve an additional reduction by about 60 %. In total this reduces the time required for the propagation of network dynamics by more than 80 % from 4g with 8 threads to 5g with 64 threads, although this was not an explicit optimization target in the design of the new kernel. At a network size of half a billion neurons, simulating 1 second of biological time requires about 30 min of wall-clock time on JUQUEEN using the 4g kernel. The new technology implemented in 5g reduces this time to about 5 min on JUQUEEN. Support for massive threading will become even more important with modern CPUs being able to run hundreds of concurrent threads.

The new technology relies on MPI_Alltoall to deliver spikes only to the ranks on which they are actually required and it potentially uses multiple communication rounds to communicate all spikes that accumulated in one communication interval. Alternatively one could use MPI_Alltoallv to allow ranks to communicate different chunk sizes to different ranks, which would require only a single communication round per communication interval. MPI_Alltoallv, however, requires the communication of the respective chunk sizes for all ranks prior to the communication of the actual data, incurring a performance penalty for large-scale simulations (Thakur et al., 2010). Due to the round-robin distribution of neurons across compute nodes and a high number of neurons per compute node, the number of spikes generated per compute node in a single communication interval is fluctuating very little. Using MPI_Alltoall with a fixed size of MPI buffers and initiating an additional communication round only when necessary is hence most likely superior to using MPI_Alltoallv, which always requires an additional communication step. While MPI_Alltoall is feasible at this scale, it relies on global communication, which means that every compute node needs to take part in every MPI communication. An alternative to this scheme would be the definition of communicators that only include a subset of nodes, thereby possibly reducing communication time. For the round-robin distribution of neurons and the random connectivity in our benchmark model it is unlikely that a randomly picked neuron has a target on a randomly picked process for simulations involving 10^5^ compute nodes (cf. section 1). But the probability that any of the neurons on a process has at least one target on a randomly picked process is still close to one; so each process needs to communicate to each other process at some point during the simulation. So far it is hence unclear how to exploit communication patterns involving only a subset of nodes, for example neighborhood collectives, to improve performance of the simulator. In particular one needs to investigate how to map the spatial structure of a neuronal network to the topology of modern HPC systems. This is not an obvious design choice as the currently employed round-robin distribution scheme is crucial for load balancing. Any non-random distribution of neurons could incur significant performance penalties due to unbalanced work load.

Regarding alternative data structures for spike routing, two other options could be considered. The sparse table essentially allows quick access to all connections originating from a single source via a globally valid index, which is the source GID. To reduce memory usage at the expense of computation time, one could replace the sparse table with a resizable array in which the containers that hold the connections are sorted by source. This resizable array would only contain entries for the sources which have local targets and would hence not scale with the total number of neurons in the network. Upon receiving a spike one could then use a binary search on this structure to access all relevant connections. Although the complexity of this operation is only ~log*N* for a sorted resizable array, it nevertheless becomes prohibitively expensive for large networks, as this search is required for every spike to be delivered. Instead of a sorted resizable array, one could use a hash map with O(1) lookup complexity. This fast access, however, comes at the expense of increased memory usage. The new technology presented here combines the lean memory usage of plain resizable arrays with fast lookup (O(1)) by employing the additional presynaptic data structure.

Future work can further improve the connection infrastructure in several aspects. One of the main drawbacks of not storing connections separated according to their source neurons is the increased size of STDP connection objects. Each of them needs to store the time of the previous spike (8 B), required to perform weight updates. Previously, this information only needed to be stored once per source neuron. This overhead leads to an increased memory footprint of connection objects for simulations in which the total number of threads is much smaller than the in-degree of a neuron (*MT* ≪ *K*). One possible remedy is to introduce a new type of spike event that not only includes information to identify the postsynaptic target, but also the time of the previous spike of the presynaptic neuron. This, however, requires changes to the presynaptic data structures responsible for buffering spikes between communication intervals.

The data structures that hold connection information are separated according to threads. In the case that neurons on different threads on the same rank receive connections from the same source, this source needs to send one spike per thread on the target rank; the number of spikes a single neuron emits therefore scales with *MT* instead of just the number of MPI processes *M*. This leads to an increase in the amount of data to be communicated and to a growth of the presynaptic data structure with the number of threads per process. To fully exploit the large number of threads supported by modern CPUs, one needs to investigate whether this strict separation of data by threads on a single rank can be relaxed.

During construction of the presynaptic data structures, the strict separation of data by threads is not respected (cf. section 3.1.2), leading to a bottleneck during collocation of connectivity data in the MPI buffers, which contributes significantly to the init time. The algorithm responsible for collocation can potentially be improved to process these data structures in parallel. Such improvements require detailed profiling of the current implementation and the design of specific thread-parallel memory access patterns for the MPI send buffer.

In total, each process needs to communicate about *KN*_*M*_ individual objects of connectivity data to all other ranks, which totals approximately 1.5 GB for a full JUQUEEN simulation. To reduce the amount of data that need to be communicated, the simulation engine can compress the information prior to the MPI call. Since compression needs to be done separately for each rank, the resulting data generally have different sizes. This requires the use of MPI_Alltoallv, because the sizes of the compressed data need to be communicated in addition to the actual data. Also on the presynaptic side, there is a potential benefit for compressing connection information, as the target lists consume a significant portion of the total available memory for large-scale simulations (cf. Figure 7; see Morrison et al., 2005 for a simple type of compression). Instead of a resizable array storing the targets of each neuron, one could use a dynamically compressing container that only decompresses target information when needed. Assuming that neurons have average firing rates of 1 Hz, the algorithm only needs information about a neuron's targets once in 1000 communication steps for a min delay of 1 ms, or, equivalently, once in 10,000 update steps for a simulation resolution of 0.1 ms. The reduction in memory usage could hence outweigh the costs of having to compress and decompress the corresponding data. To accommodate for the bursty firing of some neuron models, this container should provide efficient caching.

Synapse models that adapt their weights in response to the precise timing of pre- and postsynaptic spikes need to take into account that the spikes of pre- and postsynaptic neuron take effect on the synapse after different delays, which are referred to as axonal and dendritic delay, respectively. Different partitions of axonal and dendritic delay can result in systematic depression or systematic potentiation of synaptic weights in network simulations with STDP (Morrison et al., 2008). The new connection infrastructure is the prerequisite for the introduction of models of synaptic plasticity such as STDP with predominantly axonal delays. In previous kernel versions, only fractions of up to 50 % axonal delay can be implemented in a straight-forward fashion (see Morrison et al., 2007) as spikes are delivered through the synapses to the postsynaptic targets at the beginning of every communication interval. The contributions of the spikes are then directly incorporated in the ring-buffers of the target neurons. Axonal fractions of the delay of more than 50 % could require altering some of the spike contributions retrospectively. With the additional information on the presynaptic side, one can introduce axonal delays by postponing the time when spikes are added to the MPI send buffer. This extension requires changes to the presynaptic data structures and also entails changes to the user interface, which currently only supports the definition of synaptic transmission delays but not of an axonal fraction; by default the delay is assumed to be purely dendritic.

With the introduction of the new technology, implementations of further biophysical mechanisms such as structural plasticity and the support of continuous interactions between neurons needed to be adapted, as they are tightly coupled to the connection infrastructure. The new kernel of the NEST simulation code encompasses all biophysical mechanisms of the previous kernel.

The new technology supports the creation and deletion of synapses at runtime, which is a prerequisite for models of structural plasticity. However, since connectivity information is now spread over two data structures instead of one, an efficient implementation of particular models of structural plasticity can become more challenging. For the particular case of the structural plasticity described by Diaz-Pier et al. (2016), the new simulation kernel is slightly faster for simulations that employ less than about one hundred MPI processes (section 5.4). For larger simulations, the performance degrades faster than previously since the implementation destroys the presynaptic part whenever connections are created or removed. As this structural plasticity model is updated on a time grid much larger than the simulation resolution, typically on the order of seconds, the impact of this naive implementation on the runtime is acceptable. However, the algorithm on which the model is based is not well scalable to thousands of processes, as it collects global information about possible connections on each compute node (Diaz-Pier et al., 2016). The new two-tier connection infrastructure opens the possibility of improved implementations that do not require communication between MPI processes during structural plasticity updates and only operate on the locally available data structures. Further work is required to provide a common framework for scalable structural plasticity models.

Continuous coupling between units, for example via gap junctions, requires the communication of large amounts of data between compute nodes. The large sizes of the MPI buffers lead to significant scaling issues limiting the maximal network size that can routinely be simulated (see, for example, Figure 13 in Hahne et al., 2015). The directed communication of the new technology reduces the size of the MPI buffers. Although a detailed investigation of continuous coupling is outside the scope of the present study, directed communication potentially improves the scalability of simulations employing models with continuous interaction.

**Figure 12:**
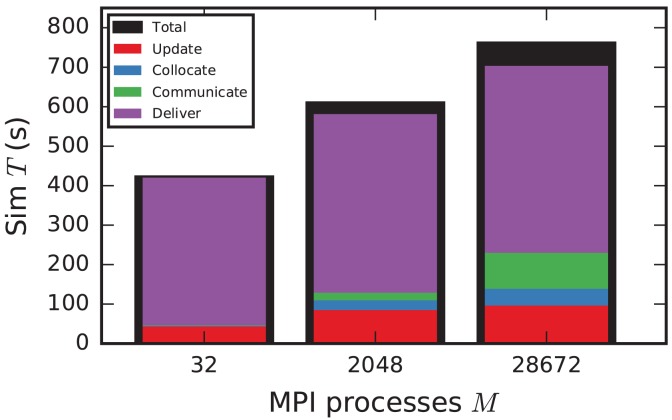
Contributions to wall-clock time for a network simulation on different numbers of MPI processes. Total wall-clock time required for propagation of network dynamics (black), time spent on update of neurons and population of spike register from target lists (red), collocation of MPI send buffer (blue), MPI_Alltoall communication (green), reading of MPI buffers and distribution of spikes via synapses to targets (purple) on the JUQUEEN supercomputer. Timings obtained via manual instrumentation of the respective parts of the source code. Same network configuration as in Figure 7.

As memory consumption is now under control for post-petascale systems, the focus of future technological developments needs to be on accelerating network construction and simulation for large-scale networks in the presence of various forms of network plasticity in order to make such simulations more attractive for neuroscientific modeling and robotics applications. Large, distributed simulations currently run at a fraction of real time, with one second of biological time requiring hundreds of seconds of compute time (Figure 12) prohibiting fast model-development cycles and studies of plasticity and learning. Most simulation time is currently spent on delivering spikes from the MPI receive buffers to the target neurons through the connection infrastructure and synapse objects (Figure 12, also see Lytton et al., 2016) and is composed of two main contributions. First, plasticity rules in their current implementation require frequent, expensive computations of exponential functions as they employ low-pass filtered spike trains. Second, spikes need to be delivered to ring-buffers of the target neurons. The latter operation requires frequent random memory access and consequently suboptimal cache usage. Performance could potentially be improved by removing the spike buffers from the individual neuron objects and using a single buffer for all spikes that need to be delivered to thread-local neurons, thereby improving memory-locality. In addition, all neuron objects are currently allocated by a single thread, which, in systems with non-uniform memory access, can lead to increased memory-access latencies (see also Ippen et al., 2017). To avoid this, neurons and connections need to be created by the thread that will most frequently access the corresponding objects. Since the contribution of delivery to the sim time is almost independent of the number of MPI processes (Figure 12), any optimization involving the delivery of spikes will have significant impact for simulations at all network scales.

For our benchmark networks the time required for communication of spike data is small compared to the time required for spike delivery (Figure 12). Since the minimal delay in the network is 1.5 ms, communication takes place on a rather coarse time grid. However, when considering other network models, especially models including randomly distributed delays (e.g., Potjans and Diesmann, 2014), the minimal delay can be of the same order of magnitude as the simulation resolution, significantly increasing the relative contribution of communication time to the total wall-clock time. To address these modeling scenarios, alternative communication schemes need to be explored that possibly interleave communication and delivery of spikes by using non-blocking MPI calls.

The network model we consider here represents a worst-case scenario in terms of connectivity, since all pairs of neurons have the same probability of forming a connection. Biological neuronal networks larger than the cortical microcircuit, however, exhibit spatial organization on multiple levels. In a local area for example, the connection probability for a pair of neurons decreases with their distance and long-range connections have a longer transmission delay than short-range connections (Hellwig, 2000; Stepanyants et al., 2009; Markov et al., 2014). Thus, at the brain scale there is indeed the biophysical basis for the idea indicated above, to map the spatial organization of structured neuronal network models (e.g., Senk et al., 2015; Schmidt et al., 2016) to the topology of the networks of HPC systems. Whether this would allow, for example, the use of MPI neighborhood collectives needs to be investigated. Kozloski and Wagner (2011) take the idea to the extreme and abandon the concept of a dynamics on a graph. They decompose neural tissue into regular blocks in three-dimensional space and allow communication only between adjacent surfaces of blocks. So far, however, it remains unclear whether a similar strategy can be used for more efficient simulations of large-scale networks of simple neuron models.

As the scale of HPC systems increases toward the exascale, so does the probability of device failure (Thakur et al., 2010; Dongarra et al., 2011; Cappello et al., 2014). To avoid wasting precious computational resources upon device failure, fault-tolerant communication options need to be explored. In particular support for malleability, the possibility of changing the number of MPI processes or threads per process during a running simulation, would improve the fault tolerance of the application upon failure of a single core and should be considered in future developments. Since for large-scale simulations, one typically uses the whole available memory per compute node, it is most likely not possible to recover from failure of a complete compute node, even with a malleable program. To cope with this type of failure, one needs to explore options for regularly storing the progress of the simulation, called checkpointing, in order to be able to restart the simulation from a given point after the crash.

While in our particular network model the propagation of single-cell dynamics only accounts for a small fraction of the total wall-clock time required for the simulation (Figure 12), this ratio can change if one considers more complex neuron models, for example models with non-linear sub-threshold dynamics or multiple compartments. To address this issue, the possibility of using accelerators available in modern HPC systems, for example general-purpose graphics processing units or field-programmable gate arrays, needs to be investigated. Here, efficient memory access of the accelerator to main memory is critical. Recent developments of the NEURON simulator core (Carnevale and Hines, 2006) include optimizations to exploit vectorization support of modern CPUs (Kumbhar et al., 2016), leading to a significant reduction of wall clock time for the investigated scenarios. It remains to be shown in how far these optimizations can be efficiently ported to other network models, simulators, and computer architectures.

The simulation technology presented in this manuscript exploits the available main memory of contemporary supercomputers and shows perfect scalability of memory usage in a weak-scaling scenario. Now the focus lies on accelerating simulations in the presence of various forms of network plasticity.

## Author contributions

All authors listed, have made substantial, direct and intellectual contribution to the work, and approved it for publication.

### Conflict of interest statement

The authors declare that the research was conducted in the absence of any commercial or financial relationships that could be construed as a potential conflict of interest. The reviewer MD declared a shared affiliation, with no collaboration, with one of the authors, SK, to the handling Editor.

## Footnotes

^1^https://www.gnu.org/software/gsl/

^2^http://nest-initiative.org/

^3^https://www.gnu.org/licenses/old-licenses/gpl-2.0.html

^4^http://www.nest-simulator.org/
