# Corrigendum: Extremely Scalable Spiking Neuronal Network Simulation Code: From Laptops to Exascale Computers

**DOI:** 10.3389/fninf.2018.00034

**Published:** 2018-07-04

**Authors:** Jakob Jordan, Tammo Ippen, Moritz Helias, Itaru Kitayama, Mitsuhisa Sato, Jun Igarashi, Markus Diesmann, Susanne Kunkel

**Affiliations:** ^1^Institute of Neuroscience and Medicine (INM-6) and Institute for Advanced Simulation (IAS-6) and JARA Institute Brain-Structure-Function Relationships (INM-10), Jülich Research Centre, Jülich, Germany; ^2^Faculty of Science and Technology, Norwegian University of Life Sciences, Ås, Norway; ^3^Department of Physics, Faculty 1, RWTH Aachen University, Aachen, Germany; ^4^Advanced Institute for Computational Science, RIKEN, Kobe, Japan; ^5^Computational Engineering Applications Unit, RIKEN, Wako, Japan; ^6^Department of Psychiatry, Psychotherapy and Psychosomatics, Medical Faculty, RWTH Aachen University, Aachen, Germany; ^7^Department of Computational Science and Technology, School of Computer Science and Communication, KTH Royal Institute of Technology, Stockholm, Sweden; ^8^Simulation Laboratory Neuroscience – Bernstein Facility for Simulation and Database Technology, Jülich Research Centre, Jülich, Germany

**Keywords:** supercomputer, large-scale simulation, parallel computing, spiking neuronal network, exascale computing, computational neuroscience

Unfortunately there was a production error in three of the illustrations of the published work that distorted several graphical elements. The correct versions of Figures 3, 5, 6 appear below. The authors apologize for the mistake. This error does not affect the quantitative displays and scientific conclusions of the article in any way.

**Figure 3:**
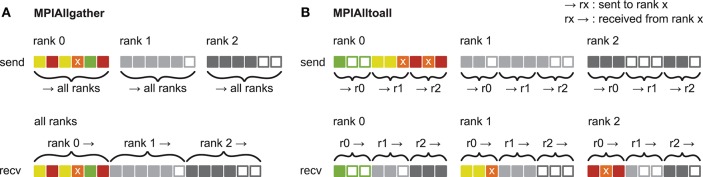
Communication of receiver-selective data using MPI_Allgather and MPI_Alltoall. The panels illustrate send and receive buffers for the example of an MPI communication that involves three ranks. Squares represent single buffer entries. Both collective MPI calls require homogeneous data types and equal send and receive buffer sizes for all ranks, which can entail sending empty buffer entries (unfilled squares). For the data that is sent by rank 0, colors indicate whether the data is required only by rank 0 (green), rank 1 (yellow), rank 2 (red), or both rank 1 and 2 (orange). For clarity, desired destinations for data that is sent by rank 1 and 2 are not indicated. **(A)** MPI_Allgather: All ranks receive the complete send buffer from all ranks, which can include unneeded data (e.g., rank 1 and 2 both receive the required orange entry but they also receive the unnecessary green entry). The receive buffer is a concatenation of all send buffers and the receive buffer size hence scales with the total number of ranks taking part in the communication. **(B)** MPI_Alltoall: Send buffers consist of equally sized sections that are destined for different receiving ranks, which allows each rank to define the data to be transmitted to any particular rank; for example, rank 0 sends the yellow entries only to rank 1. Each rank has to send identically-sized buffer sections to each rank, which can entail sending empty buffer entries or even entirely empty buffer sections. Rank 2, for example, sends an empty buffer section to rank 1. To send specific data to multiple ranks, the sending rank needs to copy the data to the send-buffer sections of all intended receiving ranks, which leads to redundancy in the send buffer; rank 0, for example, sends the orange entry “x” to both, rank 1 and 2. The size of the receive buffers is identical to the size of the send buffers and independent of the number of ranks participating in the communication.

**Figure 5:**
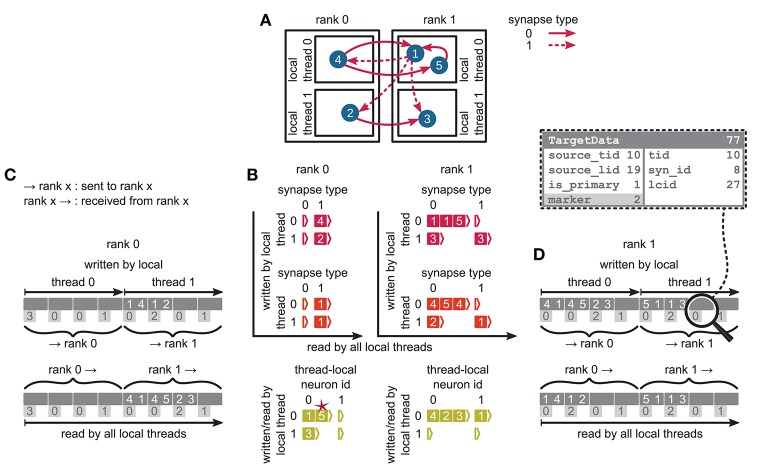
Communication of connectivity data from postsynaptic to presynaptic side for the two-tier connection infrastructure. Example network of 5 neurons **(A)** with global identifiers (GIDs) 1 to 5 (blue filled circles with white numbers) that are connected via two different types of synapses (pink arrows); for simplicity, the two types have synapse-type index 0 and 1 (solid and dashed arrows, respectively). Neurons are distributed across 2 MPI processes (outer rectangles) and 2 threads per process (inner rectangles); 4 threads in total. Synapses are hosted by the threads of their postsynaptic neurons. **(B)** From top to bottom: Connection table, source table, and target table of the example network in **(A)** on rank 0 (left) and rank 1 (right). Color code as in **Figure 4A** in Jordan et al. (2018): Synapses, sources, and targets shown as pink, red, and green filled squares, respectively, where white numbers indicate target GIDs, source GIDs, and target GIDs again, respectively. The pink star indicates redundant connection information that is absent in the optimization for small-scale simulations (cf. section 3.3 in Jordan et al., 2018). All tables are three-dimensional resizable arrays: Outermost resizable arrays for threads (vertical axes), middle resizable arrays for synapse types or local neurons (horizontal axes), innermost resizable arrays that hold the individual objects indicated by chevrons. When two neurons are connected, the thread of the postsynaptic neuron adds the new synapse to the connection table and a corresponding Source entry to the source table. Connectivity data needs to be communicated to the presynaptic side at the beginning of the simulation in order to construct the target table. **(C,D)** MPI send buffer (top) and receive buffer (bottom) that contain the TargetData of the example network, for rank 0 and rank 1, respectively; TargetData bit field shown in dashed line rectangle. Top rows (dark gray): Each field contains zero or two entries, which indicate the (source GID, target GID)-tuple. Bottom rows (light gray): Flags in each TargetData used for communication of status values among all processes (0: default, 1: no more data to send, 2: end of valid data in section, 3: skip this section).

**Figure 6:**
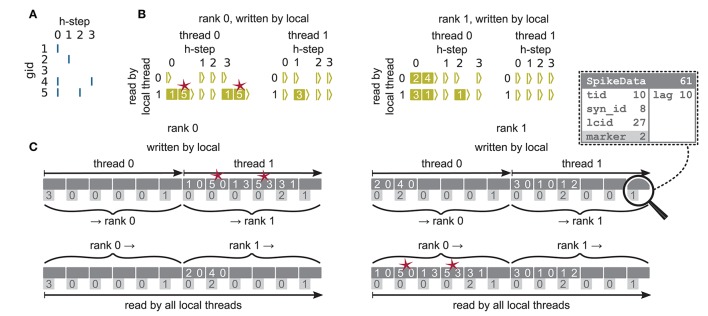
Communication of spike data using MPI_Alltoall for the example network in **Figure 5A** and an example activity **(A)**, where a communication step consists of four neuronal update steps (h-steps); spikes are shown as blue bars. **(B)** Spike register, which temporarily buffers spikes before they are collocated in the communication buffers on rank 0 (left) and rank 1 (right). Numbers indicate target GIDs. Pink stars indicate redundant information, absent in the optimization for small-scale simulations (cf. section 3.3 in Jordan et al., 2018). **(C)** MPI send buffers (top) and receive buffers (bottom) for rank 0 (left) and rank 1 (right) that contain SpikeData for the example activity. SpikeData bit fields shown in dashed rectangle. Top rows: Each field contains zero or two entries, which indicate the (target GID, lag)-tuple. Bottom rows: Flags in each SpikeData used for communication of status values among all processes (0: default, 1: no more data to send, 2: end of valid data in section, 3: skip this section).

The original article has been updated.

## Conflict of interest statement

The authors declare that the research was conducted in the absence of any commercial or financial relationships that could be construed as a potential conflict of interest.
