# Supplementary material for: Extremely Scalable Spiking Neuronal Network Simulation Code: From Laptops to Exascale Computers
Source: Front Neuroinform. 2018 Feb 16;12:2. doi: 10.3389/fninf.2018.00002 (PMC5820465; doi:10.3389/fninf.2018.00002)
Supplement: Supplementary file 1 [file Appendix.pdf]

Appendix for: "Extremely scalable spiking neuronal network simulation code: from laptops to exascale computers"

Jakob Jordan [1] Tammo Ippen [1,2] Moritz Helias [1,3]
Itaru Kitayama[4] Mitsuhisa Sato[4] Jun Igarashi[5]
Markus Diesmann [1,3,6] Susanne Kunkel [7,8]

February 4, 2018

[1] Institute of Neuroscience and Medicine (INM-6) and Institute for Advanced Simulation (IAS-6) and JARA Institute Brain Structure Function Relationship (INM-10), Jülich Research Centre, Jülich, Germany
[2] Faculty of Science and Technology, Norwegian University of Life Sciences, Ås, Norway
[3] Department of Physics, Faculty 1, RWTH Aachen University, Aachen, Germany
[4] Advanced Institute for Computational Science, RIKEN, Kobe, Japan
[5] Computational Engineering Applications Unit, RIKEN, Wako, Japan
[6] Department of Psychiatry, Psychotherapy and Psychosomatics, Medical Faculty, RWTH Aachen University, Aachen, Germany
[7] Department of Computational Science and Technology, School of Computer Science and Communication, KTH Royal Institute of Technology, Stockholm, Sweden
[8] Simulation Laboratory Neuroscience — Bernstein Facility Simulation and Database Technology, Institute of Advanced Simulations, Jülich Research Centre and JARA, Jülich, Germany

A Network and simulation parameters

Table 1, Table 2 and Table 3 summarize the network model and parameters.

A: Model Summary	
**Populations**	Two: excitatory, inhibitory
**Topology**	–
**Connectivity**	Random convergent connections
**Neuron models**	Leaky integrate-and-fire (LIF), fixed firing threshold, fixed absolute refractory time (voltage clamp), α-currents
**Channel models**	–
**Synaptic plasticity**	Spike-timing dependent plasticity at excitatory-excitatory recurrent connections
**External input**	Independent fixed-rate Poisson spike trains to all neurons
**Measurements**	Total number of spikes generated per process
**Other**	Multapses allowed, no autapses

B: Populations		
**Name**	**Elements**	**Size**
E	LIF neuron	$N_\mathrm{E} = 4N_\mathrm{I}$ with $N_\mathrm{E} + N_\mathrm{I} = N$
I	LIF neuron	N_I

C: Connectivity			
**Name**	**Source**	**Target**	**Pattern**
EE	E	E	Random convergent, $K_\mathrm{E} \to 1$, weight variable, delay d
IE	E	I	Random convergent, $K_\mathrm{E} \to 1$, weight J, delay d
EI	I	E	Random convergent, $K_\mathrm{I} \to 1$, weight $-gJ$, delay d
II	I	I	Random convergent, $K_\mathrm{I} \to 1$, weight $-gJ$, delay d

Table 1: Tabular description of network model after Nordlie et al. (2009).

D: Neuron Model	
**Name**	LIF neuron
**Type**	Leaky integrate-and-fire, α-current input
**Subthreshold dynamics**	$\tau_m \frac{dV(t)}{dt} = -V(t) + \frac{I(t)}{C_m}$ if $(t > t^* + \tau_{ref})$<br>$V(t) = V_{\text{reset}}$ else
**Synaptic current dynamics**	For each presynaptic spike:<br>$I_{\text{syn}}(t) = w \frac{e}{\tau_{\text{syn}}} t\, e^{-t/\tau_{\text{syn}}}$
**Spiking**	If $V(t-) < \Theta$ and $V(t+) \geq \Theta$<br>1. set $t^* = t$<br>2. emit spike with time stamp t^*

E: Synapse Model	
**Name**	Power law STDP (Morrison et al., 2007)
**Type**	Weight dependent STDP with a power law update rule for potentiation and a multiplicative update rule for depression
**Spike pairing scheme**	All-to-all (for nomenclature see Morrison et al., 2008)
**Pair-based update rule**	$\Delta w^+ = F_+(w)\, e^{-\frac{\vert\Delta t\vert}{\tau_+}}$ if $\Delta t > 0$<br>$\Delta w^- = -F_-(w)\, e^{-\frac{\vert\Delta t\vert}{\tau_-}}$ else<br>Δt - temporal difference between post- and pre-synaptic spikes, synaptic delay considered to be purely dendritic
**Weight dependence**	$F_+(w) = \lambda w_0^{1-\mu} w^{\mu}, \quad F_-(w) = \lambda \alpha w$

F: Input		
**Type**	**Target**	**Description**
**Poisson generator**	I&E	Independent for each neuron, rate $\nu_{\text{x}} K_{\text{x}}$, weight J

Table 2: Tabular description of network model after Nordlie et al. (2009), continued.

B: Populations		
**Name**	**Value**	**Description**
N_E	variable	Size of excitatory population E
N_I	variable	Size of inhibitory population I

C: Connectivity		
**Name**	**Value**	**Description**
K_E	9000	Number of incoming connections from E
K_I	2250	Number of incoming connections from I

D: Neuron Model		
**Name**	**Value**	**Description**
τ_m	10 ms	Membrane time constant
C_m	250 pF	Membrane capacitance
Θ	20 mV	Fixed firing threshold
V_0	0 mV	Resting potential
V_{reset}	V_0	Reset potential
τ_{ref}	0.5 ms	Absolute refractory period
τ_{syn}	0.33 ms	Rise time of PSC
μ_V	5.7 mV	Mean value of initial normal distribution of membrane potentials
σ_V	7.2 mV	Standard deviation of initial normal distribution of membrane potentials

E: Synapse Model		
**Name**	**Value**	**Description**
τ_+	15 ms	Time constant of potentiation window
τ_-	30 ms	Time constant of depression window
λ	0.1	Learning rate
μ	0.4	Weight-dependence parameter of potentiation
α	0.0513	Asymmetry parameter
w_0	1 pA	Normalization parameter
d	1.5 ms	Delay
J	0.14 mV	Excitatory weight
g	5.0	Relative inhibitory weight

F: Input		
**Name**	**Value**	**Description**
K_x	9000	Number of external inputs
ν_x	$2.3\,\frac{\text{spikes}}{\text{s}}$	Rate of single external input

Table 3: Simulation parameters after Nordlie et al. (2009).

B Connection infrastructure for devices

Next to the neuronal network, a virtual experiment requires representations of further components. For example, an electrophysiological experiment may

encompass a current generator for the stimulation of individual neurons and a multi-channel amplifier for the acquisition of the membrane potential of multiple neurons. In the concept implemented in the NEST software (Gewaltig and Diesmann, 2007) these entities are additional nodes of the network collectively referred to as 'devices'. Although a primary motivation is to not only formally describe the system under study but also the measurement process, also devices without a real-world counterpart can be defined. These are useful, for example, for obtaining the time course of an arbitrary state variable of an abstract neuron model. To avoid extensive communication between compute nodes, stimulation and recording devices should be co-located on the same compute node as the neurons to which they are sending events and from which they are receiving events, respectively. In NEST, creation of a single stimulation or recording device leads to a creation of a device object on every thread. These device objects only send data to and receive data from neurons located on the same thread. This has two advantages: On the one hand, processes do not need to exchange data that is sent from and received by devices, and on the other hand this supports parallel post-processing of recorded data, since the data is already distributed over compute nodes. To simplify maintenance and construction of the two-tier connection infrastructure managing connections between neurons, connections to and from devices are stored in separate data structures that consist of two four-dimensional resizable arrays (Figure 1). Connections from devices are stored according to (i) the thread of the device, (ii) the thread-local id of the device, and (iii) the synapse type. If a device sends an event (e.g., a spike generated by a Poisson generator), it is delivered through all connections for all synapse types to the corresponding targets. Connections to devices are stored according to (i) the thread of the sending neuron, (ii) the thread-local id of the neuron and (iii) the synapse type. If a neuron sends an event, it is delivered through all connections for all synapse types to the corresponding target devices. Due to their small memory footprint, these data structures are not included in the memory model.

The separate data structures for connections to and from devices can improve the delivery of data from devices to neurons, for example from Poisson sources, with regard to the previous kernel. While the previous technology uses the same infrastructure employed by neurons to communicate spikes, devices have now a more direct access to their targets.

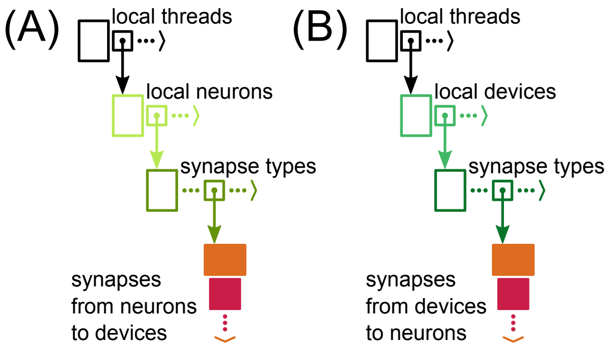

Figure 1: **Connection infrastructure for devices of the NEST 5g kernel.** **(A)** Connections from neurons to thread-local devices. Each MPI process stores a pointer to a dynamic container of connection objects for every thread, every thread-local neuron, and every synapse type. If a synapse type is in use, the corresponding container (orange filled rectangle) holds the synapses objects (pink filled squares). **(B)** Connections from devices to thread-local neurons. A data structure of identical shape is used to store the connections from devices to thread-local neurons.

C Scalability of `MPI_Allgather` and `MPI_Alltoall`

Here we investigate the scaling properties of `MPI_Allgather` and `MPI_Alltoall` on JUQUEEN for increasing numbers of MPI processes and MPI send buffer sizes. In network simulators, naively switching from a source-based AER using `MPI_Allgather` to a target-based AER scheme using `MPI_Alltoall` would lead to an average increase of the send buffer size proportional to the average out-degree of neurons independent of the number of processes, as in the case of 5g-nosort. Here we assume that the out-degree is $K = 10,000$ and hence increase the send buffer size for all `MPI_Alltoall` measurements correspondingly. We measure the average time a single MPI communication among M nodes takes for an MPI send buffer of size s. In addition we measure the latency by setting the send buffer size to zero.

For `MPI_Allgather`, the latency is almost constant, independent of the number of processes. For non-empty send buffers however, the wall-clock time of a single MPI call increases significantly with the number of processes, almost linearly for large send buffer sizes (Figure 2). Even though the send buffers are very small, the size of receive buffers scales linearly in the number of MPI processes. The increase in communication time for `MPI_Allgather` is most likely

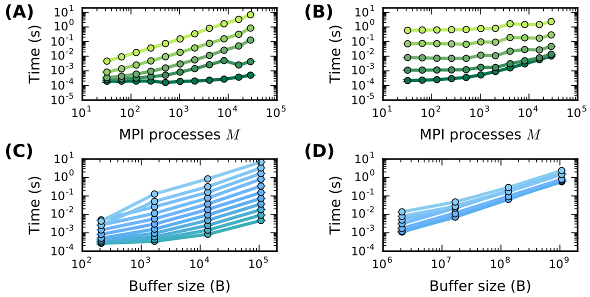

Figure 2: **Scaling of communication time for `MPI_Allgather` (left) and `MPI_Alltoall` (right) over MPI processes (top) and send buffer sizes (bottom).** Communication time measured on JUQUEEN. Darker colors indicate smaller buffer sizes (top) and smaller number of MPI processes (bottom), respectively. Number of MPI processes $M \in 32; 64; 128; 256; 512; 1024; 2048; 4096; 8192; 16,384; 28,672$. Send buffer sizes for `MPI_Allgather` $s \in 0\,\text{kB}; 0.2\,\text{kB}; 1.6\,\text{kB}; 13\,\text{kB}; 105\,\text{kB}$, for `MPI_Alltoall` $s \in 0\,\text{kB}; 2048\,\text{kB}; 16,384\,\text{kB}; 131,072\,\text{kB}; 1,048,576\,\text{kB}$. Error bars denote SEM and are partly covered by markers.

due to the increase in receive buffer sizes that require an increasing amount of memory to be written. For `MPI_Alltoall`, on the other hand, latency increases significantly with the number of processes while scaling of communication time for non-empty buffers is much better than for `MPI_Allgather`, with only a small increase over the range of investigated processes. Note that the total size of the send buffers is much larger for `MPI_Alltoall` with a maximal send buffer size of 1 GB that is communicated across 28,672 compute nodes in ~ 2 s. From 10^4 processes on, the receive buffers of `MPI_Allgather` are larger than for `MPI_Alltoall`, leading to a better performance of `MPI_Alltoall` (Figure 2**A**,**B**). When scaling the buffer size, communication time increases supra-linearly for `MPI_Allgather`, while it increases almost linearly for `MPI_Alltoall`, almost independent of the number of MPI processes (Figure 2**C**,**D**).

D Structural plasticity

Structural plasticity modifies the connectivity of the network at runtime, for example allowing researchers to grow network connectivity according to certain rules instead of specifying it at the beginning of the simulation (Diaz-Pier et al., 2016). Due to the purely postsynaptic storage of connections in the previous

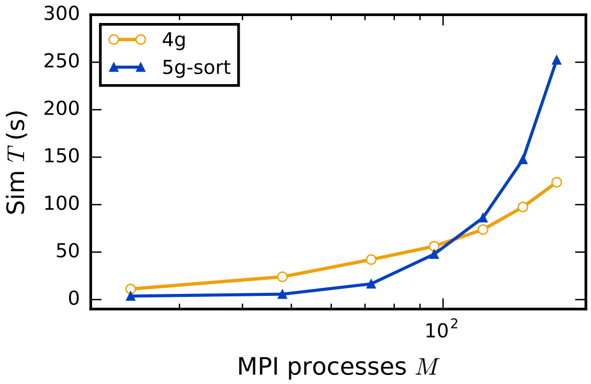

Figure 3: **Weak scaling of a neuronal network simulation with structural plasticity on JURECA.** Runtime for simulating 5000 neurons per compute node for 10 s of biological real time using one thread per MPI process. $M \in 24; 48; 72; 96; 120; 144; 168$. Same line styles and color code as in Figure 7 in the main text.

simulation kernel, structural plasticity only needed to manipulate postsynaptic data structures. However, due to the two-tier connection infrastructure in the new kernel, it becomes necessary to update also the presynaptic part when connections are created or removed at runtime. Since the scalability of the structural plasticity algorithm in the previous kernel is only feasible up to a few hundreds of processes, we provide only a naive implementation for the new simulation kernel. In this implementation, the whole presynaptic part of the connection infrastructure is destroyed whenever new connections are created or connections are removed and then reconstructed from scratch from the postsynaptic part. While this is a fairly expensive operation, structural plasticity operates on slow timescales in the seconds range, making this approach feasible up to about $\mathcal{O}(100)$ MPI processes. In this range the performance is comparable to the previous simulation kernel Figure 3. To support larger simulations, an alternative implementation needs to be developed, that in the optimal case operates only on local data structures, without requiring global communication among all compute nodes.

E Strong scaling over threads

Modern simulation codes need to be able to exploit the increased parallelism available on compute nodes that support tens to hundreds of concurrent threads. Here we investigate strong scaling over threads for the previous and the new kernel. For an increasing number of threads the build time scales very well for both kernel versions, as long as the number of threads is smaller or equal to the number of available cores (Figure 4). As soon as hyper-threading is used, the scaling behavior becomes slightly worse, leading to smaller differences between 32 and 64 threads. As nodes are constructed serially, this contribution does not scale over threads but is in any case negligible compared to the time spent on the construction of connections. The init time does not scale with the number of threads for the new kernel and even increases for a large number of threads that exceeds the core count. As in the weak scaling scenario, most of the time is spent on collocating MPI buffers which does not significantly profit from multiple threads and is mainly limited by memory access not processing speed. The sim time decreases monotonously for the new kernel, even beyond 16 threads in contrast to the old kernel. For both kernels, the memory consumption increases with the number of threads, not allowing simulations with more than 16 threads when employing the old kernel. Further detailed investigations are necessary to improve scaling of the init time with an increasing number of threads in the new kernel.

F Strong scaling over MPI processes (full data)

Figure 5 contrasts build time, init time, and memory usage with the simulation time already shown in Figure 9 in the main text.

References

Diaz-Pier, S., Naveau, M., Butz-Ostendorf, M., and Morrison, A. (2016). Automatic generation of connectivity for large-scale neuronal network models through structural plasticity. *Front. Neuroanat.* 10, 57. doi:10.3389/fnana.2016.00057

Gewaltig, M.-O. and Diesmann, M. (2007). NEST (NEural Simulation Tool). *Scholarpedia* 2, 1430. doi:10.4249/scholarpedia.1430

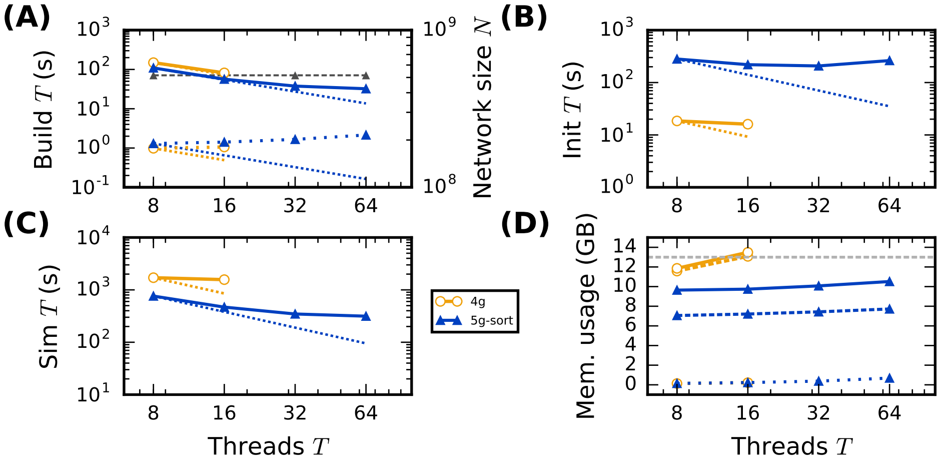

Figure 4: **Strong scaling over threads of an archetypal neuronal network simulation on a petascale computer.** Runtime and memory usage per compute node for an increasing number of threads per MPI process ($T \in$ 8; 16; 32; 64) and $M = 28,672$ MPI processes on JUQUEEN. Other parameters same as in Figure 7 in the main text. Solid lines indicate decrease of the respective times for perfect scaling. Same line styles and color code as in Figure 7 in the main text.

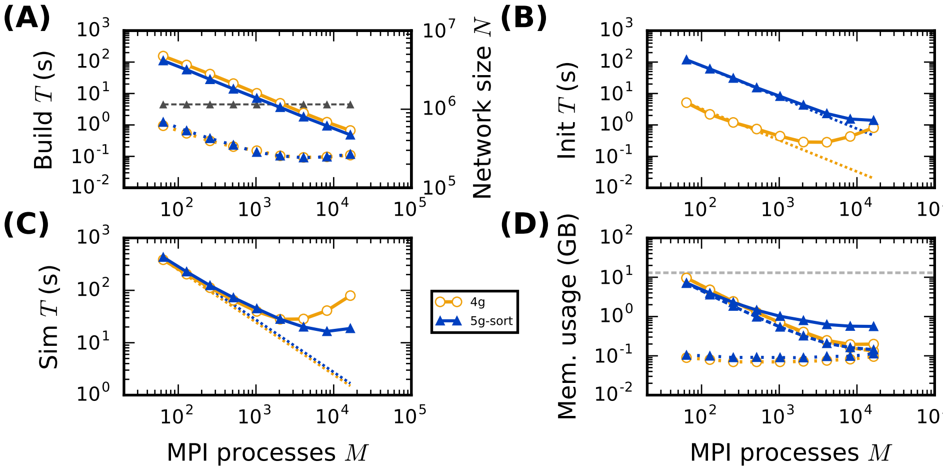

Figure 5: **Strong scaling of an archetypal neuronal network simulation on a petascale computer.** Data obtained from JUQUEEN. Same line styles and color code as in Figure 7 in the main text.

Morrison, A., Aertsen, A., and Diesmann, M. (2007). Spike-timing dependent plasticity in balanced random networks. *Neural Comput.* 19, 1437–1467. doi:10.1162/neco.2007.19.6.1437

Morrison, A., Diesmann, M., and Gerstner, W. (2008). Phenomenological models of synaptic plasticity based on spike-timing. *Biol. Cybern.* 98, 459–478. doi:10.1007/s00422-008-0233-1

Nordlie, E., Gewaltig, M.-O., and Plesser, H. E. (2009). Towards reproducible descriptions of neuronal network models. *PLoS Comput. Biol.* 5, e1000456. doi:10.1371/journal.pcbi.1000456
